# Dynamic predictions using flexible joint models of longitudinal and time‐to‐event data

**DOI:** 10.1002/sim.7209

**Published:** 2017-01-22

**Authors:** Jessica Barrett, Li Su

**Affiliations:** ^1^ Strangeways Research Laboratory, Department of Public Health and Primary CareUniversity of CambridgeWorts CausewayCambridgeCB1 8RNU.K.; ^2^ MRC Biostatistics UnitSchool of Clinical Medicine, University of Cambridge Robinson WayCambridgeCB2 0SRU.K.

**Keywords:** P‐splines, random effects, shared parameter models, survival analysis

## Abstract

Joint models for longitudinal and time‐to‐event data are particularly relevant to many clinical studies where longitudinal biomarkers could be highly associated with a time‐to‐event outcome. A cutting‐edge research direction in this area is dynamic predictions of patient prognosis (e.g., survival probabilities) given all available biomarker information, recently boosted by the stratified/personalized medicine initiative. As these dynamic predictions are individualized, flexible models are desirable in order to appropriately characterize each individual longitudinal trajectory. In this paper, we propose a new joint model using individual‐level penalized splines (P‐splines) to flexibly characterize the coevolution of the longitudinal and time‐to‐event processes. An important feature of our approach is that dynamic predictions of the survival probabilities are straightforward as the posterior distribution of the random P‐spline coefficients given the observed data is a multivariate skew‐normal distribution. The proposed methods are illustrated with data from the HIV Epidemiology Research Study. Our simulation results demonstrate that our model has better dynamic prediction performance than other existing approaches. © 2017 The Authors. *Statistics in Medicine* Published by John Wiley & Sons Ltd.

## 1 Introduction

In many clinical trials and observational studies, longitudinal biomarker information is often collected together with information on a time‐to‐event outcome (e.g., patient survival). Joint modeling is becoming increasingly popular in characterizing the coevolution of the longitudinal and time‐to‐event processes 1. Recently boosted by the stratified/personalized medicine initiative, a cutting‐edge research direction in the joint modeling area is individualized dynamic predictions of patient prognosis (e.g., survival probabilities) using all available biomarker information. Pioneer works include Yu *et al*. 2, Proust‐Lima and Taylor 3, Rizopoulos 4, and Taylor *et al*. 5.

As dynamic predictions for patient prognosis are individualized, flexible joint models are desirable in order to appropriately characterize the longitudinal process for each individual. In this paper, we propose a new flexible joint model with individual‐level penalized splines (P‐splines) 6 to characterize the coevolution of the longitudinal and time‐to‐event processes. One important strength of our model is that predicting survival probabilities becomes straightforward because the posterior distribution of individual‐level (random) P‐spline coefficients is a multivariate skew‐normal distribution. We will start by reviewing relevant literature on flexible joint models, and then, we will describe the main idea of our approach, followed by details of our data example.

### 1.1 Flexible joint models

In existing joint models of longitudinal and time‐to‐event (survival) data, it is often assumed that individual longitudinal trajectories are characterized by a linear model with random intercepts and random time slopes 7. However, in long‐term follow‐up studies, individual longitudinal trajectories may not follow this simple linear model, which makes it challenging to examine the associated evolutions of the longitudinal and time‐to‐event processes. To overcome this problem, Brown *et al.* 8 proposed a flexible Bayesian B‐spline model for the longitudinal process; Ding and Wang 9 also developed a nonparametric multiplicative random effects model to flexibly model the longitudinal process. More recently, Rizopoulos and Ghosh 10 proposed a Bayesian semiparametric multivariate joint model for multiple longitudinal outcomes and a time to event and discussed various parameterizations in the survival sub‐model. Non‐spline‐based methods such as fractional polynomials have also been developed 11, 12, where the survival sub‐model was based on cumulative hazard. None of the aforementioned works focused on dynamic predictions. Recently, Rizopoulos [7, Chapter 7] exemplified dynamic predictions based on a flexible joint model using B‐splines with one interval knot in the longitudinal sub‐model, but the smoothness (degree of freedom) of the B‐splines was predefined and not chosen based on the data. Overall, frequentist approaches based on maximum likelihood estimation to flexible modeling of the longitudinal process in the joint modeling setting are still limited because of computational cost and complexity 13.

Recently, Barrett *et al.* 14 developed a joint model that allows more flexible random effect structures in the longitudinal sub‐model. The key idea in their development was to discretize the time scale of the time‐to‐event outcome and let separate random effects within the discrete time intervals in the longitudinal sub‐model (i.e., time‐dependent random effects) be associated with the hazards of event occurrence in the corresponding time intervals. For example, a stationary Gaussian process was assumed for their random effect model. However, their specification of time‐dependent random effects is somewhat limited by the fact that only a single random effect is used to characterize the evolution of the longitudinal process within a discrete time interval and the serial correlation between random effects is assumed to be stationary over time. Barrett *et al.* 14 did not investigate dynamic predictions based on their model.

### 1.2 Joint modeling with time‐dependent random effects

In this paper, we propose a new flexible model for jointly modeling the longitudinal and time‐to‐event processes. The main idea is to use time‐dependent random effects with non‐stationary covariance structure, constructed by P‐splines 15, to model time trends in each individual longitudinal trajectory. Specifically, building upon the model in 14, we use P‐splines with a truncated linear basis 6 to model individual longitudinal trajectories, while population‐level longitudinal trajectories can be modeled by P‐splines with possibly different bases and knots. The smoothing parameters of both population‐level and individual‐level P‐splines are chosen from the data in order to avoid over‐fitting. Knots for the individual‐level P‐splines are located at the interval boundaries of the discretized time scale in order for the association between the longitudinal and survival sub‐models to be easily interpreted. In this case, the individual‐level P‐spline coefficients act as shared parameters and are used to construct time‐dependent random effects that represent the deviations of the intercepts and slopes of the individual longitudinal trajectories from the population‐level trajectory within the corresponding discrete time interval, which is different from the single random effect setup used in 14. Moreover, the covariance structure for these time‐dependent random effects are non‐stationary over time, unlike the stationary covariance structure used in 14.

### 1.3 Dynamic predictions

Model flexibility is even more important when performing individualized dynamic predictions. In practice, a simple linear model may fail to capture the nonlinear patterns in individual longitudinal trajectories for some patients, even if this model applies to the population‐level longitudinal trajectories and the majority of individual longitudinal trajectories. Because dynamic predictions are individualized and need to appropriately account for possible nonlinearity in each individual longitudinal trajectory, flexible joint modeling approaches are certainly desirable. However, too much flexibility can also be harmful because of the danger of extrapolation 16. In Section 5, we will use simulations to compare the dynamic prediction performance of our model with two other joint models with different degrees of flexibility.

A notable feature of our approach is that individualized dynamic predictions for survival probabilities over time are straightforward because under the proposed model the posterior distribution of the random P‐spline coefficients given the observed data is a multivariate skew‐normal distribution. This facilitates individualized predictions of survival probabilities 4 because no approximation (e.g., using Metropolis–Hastings algorithm) is needed to sample from this posterior distribution.

### 1.4 Example

**Figure 1:**
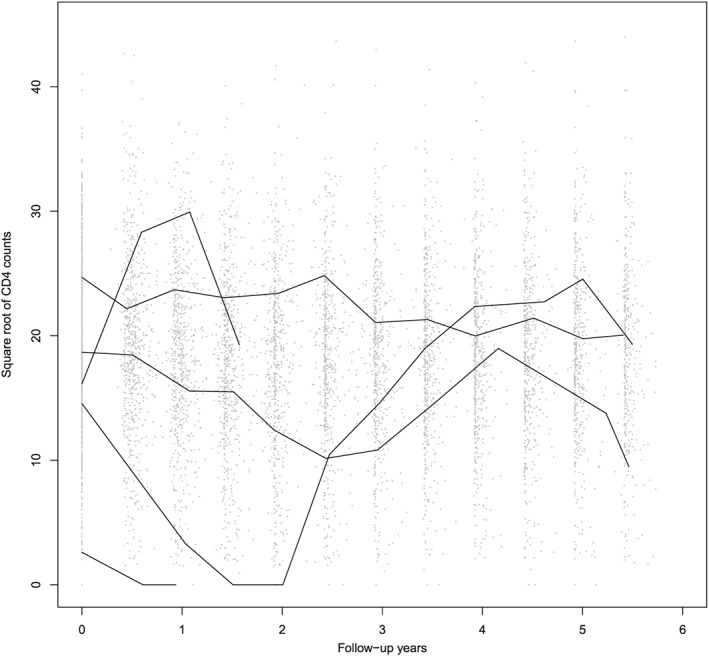
Observed (square root) longitudinal CD4 count data from the HIV Epidemiology Research Study with profiles from five selected participants highlighted.

This work is exemplified by data from the HIV Epidemiology Research Study (HERS) 17, with the aim of predicting HIV‐related survival probabilities over time by jointly modeling longitudinal CD4 count process that reflected HIV disease progression in the study population. The HERS was a longitudinal study of 1310 women with, or at high risk for, HIV infection from 1993 to 2000. During the study, 12 visits were scheduled, where a variety of clinical, behavioral, and sociological outcomes were recorded approximately every 6 months. We will focus on the 850 women who were HIV positive at enrollment, and Figure 1 plots their observed CD4 count data over time (a square root transformation is used to reduce the right skewness in these data). It is clear that some individual longitudinal CD4 count trajectories had strong nonlinear patterns, which might be explained by the fact that the highly active antiretroviral therapy (HAART) was first introduced to the HERS population around 1997 and changed the disease progression dramatically. This phenomenon therefore motivates us to build a more flexible joint model to characterize individual longitudinal trajectories in order to improve predictions of HIV‐related survival probabilities.

The rest of the paper is organized as follows. In Section 2, we introduce the proposed joint model. Estimation is described in Section 3, including the procedure for dynamic predictions. In Section 4, we apply the proposed methods to the HERS data. Simulation results are summarized in Section 5, and we conclude with a discussion in Section 6.

## 2 Joint model

Suppose that *N* independent patients are to be followed up over time. For the *i*th (*i* = 1,…,*N*) patient, there is a longitudinal outcome process {*Y*_*i*_(*t*)}, where t∈T=[0,T] is the time since enrollment and the constant *T* is determined by the potential maximum follow‐up time where a longitudinal measurement can be taken in the study. Correspondingly, there is a *p*‐dimensional covariate process {**x**_*i*_(*t*)} associated with {*Y*_*i*_(*t*)}. We assume {*Y*_*i*_(*t*)} is a continuous‐time Gaussian process with a mean function *μ*_*i*_(*t*) that is dependent on **x**_*i*_(*t*) and a variance–covariance function $\text{cov}\{Y_{i}(t),Y_{i}(t^{\prime })|x_{i}(t),x_{i}(t^{\prime })\}\;=\;V_{i}(t,t^{\prime })$( $t\;⩽\;t^{\prime }$). Parametric forms can be used for $V_{i}(t,t^{\prime })$, for example, $V_{i}(t,t^{\prime })\;=\;\sigma_{\varepsilon }^{2}I(t\;=\;t^{\prime })$ with I(·) as an indicator function.

At the same time, a time‐to‐event outcome *S*_*i*_ is being observed and the occurrence of the event terminates the observation of {*Y*_*i*_(*t*)}. Instead of using the continuous time scale T from the longitudinal process, we assume a discrete time scale S={1,2,…,M} for this time‐to‐event outcome. However, it is assumed that there is a surjection *s*(*t*) from T to S, for example, S might be a partition of T. Then, S is considered to be a series of time intervals. In the HERS example presented in Section 4, we partition the longitudinal measurement time by 6‐month intervals and determine the occurrence of HIV‐related deaths in these intervals. Further, let *C*_*i*_ be the potential censoring time for the *i*th patient. The observed event time is $S_{i}^{*}\;=\;\min(S_{i},C_{i})$, and the indicator for event occurrence is $\delta_{i}=I(S_{i}⩽C_{i})$. At continuous time points $t_{i1},\ldots ,t_{in_{i}}(t_{in_{i}}\;⩽\;S_{i}^{*})$, we also observe the longitudinal measurements $Y_{i}\;=\;{\left\{Y_{i}(t_{i1}),\ldots ,Y_{i}(t_{in_{i}})\right\}}^{T}$.

### 2.1 Longitudinal sub‐model

We assume the following model for the mean function *μ*_*i*_(*t*) of {*Y*_*i*_(*t*)}:

(1) $$\mu_{i}(t)\;=\;x_{i}{\left(t\right)}^{T}\alpha \;+\;m_{i}(t),$$

where ***α*** is a *p* × 1 vector of regression coefficients associated with covariates **x**_*i*_(*t*) and *m*_*i*_(*t*) is the true underlying time trajectory for the *i*th patient after adjusting for **x**_*i*_(*t*).

Following 6, *m*_*i*_(*t*) can be modeled by P‐splines as follows:

(2) $$m_{i}(t)\;=\;\overset{M}{\underset{l\;=\;0}{\sum }}(\beta_{l}\;+\;b_{il})B_{l}(t),$$

where {*B*_*l*_(*t*)} = {1,*t*,(*t* − *k*_1_)_ + _,…,(*t* − *k*_*M* − 1_)_ + _} is the truncated linear basis with internal knots at *k*_1_,…,*k*_*M* − 1_ on [0,*T*] and ${\left(a\right)}_{+}\;=\;a\;\cdot \;I(a\;⩾\;0)$. The location of the knots *k*_1_,…,*k*_*M* − 1_ is determined by the boundary points of S, the time intervals defined in the survival sub‐model; thus, the number of internal knots is *M* − 1. ***β*** = (*β*_0_,*β*_1_,…,*β*_*M*_)^T^ is the vector of P‐spline coefficients that characterize the population‐level longitudinal trajectory; **b**_*i*_ = (*b*_*i*0_,*b*_*i*1_,…,*b*_*iM*_)^T^ is the vector of random P‐spline coefficients that determine the deviations of individual longitudinal trajectories from the population‐level trajectory.

Note that we choose to use the truncated linear basis {*B*_*l*_(*t*)} because the time‐dependent random effects constructed by P‐spline coefficients and a truncated linear basis are easy to interpret when incorporated in the survival sub‐model. For instance, in the HERS example in Section 4, we obtain the random intercept *W*_*r*0_(**b**_*i*_) at the *r*th interval (*r* = 1,…,*M*) as

(3) $$W_{r0}(b_{i})=\left\{\begin{array}{cc} b_{i0} & r=1\\ b_{i0}\;+\;b_{i1}k_{1} & r\;=\;2\\ b_{i0}\;+\;b_{i1}k_{r\;-\;1}\;+\;\cdots \;+\;\overset{r\;-\;1}{\underset{l\;=\;2}{\sum }}b_{i,l}(k_{r\;-\;1}\;-\;k_{l\;-\;1}) & r\;=\;3,\ldots ,M \end{array}\right.$$

and the random slope *W*_*r*1_(**b**_*i*_) at the *r*th interval as

(4) $$W_{r1}(b_{i})\;=\;b_{i1}\;+\;\cdots \;+\;b_{ir};$$

see Figure S1 in the Supporting Information for a graphical illustration. In a similar manner, we have the population‐level intercept and slope at the *r*th interval *W*_*r*0_(***β***) and *W*_*r*1_(***β***); therefore, the intercept and slope of *m*_*i*_(*t*) at the *r*th interval are *m*_*i*_(*k*_*r* − 1_) = *W*_*r*0_(***β***) + *W*_*r*0_(**b**_*i*_) and *m* *i* *′*(*k*_*r* − 1_) = *W*_*r*1_(***β***) + *W*_*r*1_(**b**_*i*_), respectively. Note that *k*_0_ = 0 and *k*_1_,…,*k*_*M* − 1_ are the internal knots of the P‐splines. In the corresponding survival sub‐model, *m*_*i*_(*k*_*r* − 1_) and *m* *i* *′*(*k*_*r* − 1_) are included as time‐varying covariates and their regression coefficients represent the associations of the level of CD4 count at the beginning of the time intervals and the progression rate of CD4 count within the time intervals with the corresponding conditional survival probabilities in these intervals.

In practice, a more complex basis such as B‐splines or low‐rank thin‐plate splines (with better numerical properties) could also be used for the population‐level trajectory 15; the penalized likelihood estimation procedure in Section 3 can easily be adapted to use any basis for fitting the population‐level trajectory. For individual trajectories, because in most applications the number of data points for each patient is not large, the truncated linear basis is generally flexible enough to characterize the essential patterns of each individual longitudinal trajectory.

For both population and individual‐level P‐splines with truncated linear basis in our model, knot locations could be chosen to lie anywhere on the continuous time scale. In this paper, we choose to fix the knot locations at the discretization points in the survival sub‐model because of the following reasons. First, in practice, a summary of the rate of change of the longitudinal outcome across a discrete time interval is needed to define an association of the longitudinal outcome with the survival process. The association between the longitudinal and survival processes is therefore easier to interpret if knots lie on discrete time interval boundaries because the time slope of an individual's longitudinal trajectory is then *constant* within the time intervals given our setup of P‐splines with truncated linear basis. Second, using the sample quantiles of the longitudinal measurement times in the observed data to define the knot locations, as is common practice in semiparametric regression literature 15, could be problematic in our scenario because the knots would be closer together at earlier follow‐up times because of selection bias by the survival outcome. Because the longitudinal sub‐model is intended to characterize the longitudinal process if no truncation of the survival outcome occurs and the joint modeling approach is adopted to correct the selection bias due to the survival outcome, we choose the knot locations and discretization points according to the planned longitudinal measurement schedule, thereby avoiding dependence of the longitudinal sub‐model specification on the observed survival outcomes.

### 2.2 Survival sub‐model

Following 14, we assume a probit model for the discrete hazard rate of the event $\lambda_{ir}\;=\;P(S_{i}\;=\;r|S_{i}\;⩾\;r)$ in the *r*th interval (*r* = 1,…,*M*),

(5) $$\lambda_{ir}\;=\;1\;-\;\Phi \left({\tilde{x}}_{ir}^{T}\tilde{\alpha }\;+\;\gamma_{r}^{T}W_{ir}b_{i}\right),$$

where Φ(·) is the standard normal cumulative distribution function, ${\tilde{x}}_{ir}$ is a $\tilde{q}\;\times \;1$ vector of covariates (possibly time varying) with regression coefficients $\tilde{\alpha }$. **W**_*ir*_ **b**_*i*_ is a *q* × 1 vector of linear combinations of **b**_*i*_ (e.g., in the HERS example, we have **W**_*ir*_ **b**_*i*_ = (*m*_*i*_(*k*_*r* − 1_),*m* *i* *′*(*k*_*r* − 1_))^T^ and *q* = 2) and ***γ***_*r*_ is an association parameter vector that relates the longitudinal and time‐to‐event processes.

Depending on the applications, various parameterizations for **W**_*ir*_ **b**_*i*_ can be used; see discussions in 7 and 10. For example, we use *m*_*i*_(*k*_*r* − 1_) and *m* *i* *′*(*k*_*r* − 1_) in the HERS example in Section 4, as it is believed that the survival probabilities depend on the disease progression, but we only allow disease progression up to the end of the *r*th interval to be associated with the survival probability at the *r*th interval. Interactions between *m*_*i*_(*k*_*r* − 1_),*m* *i* *′*(*k*_*r* − 1_) and baseline covariates could also be included.

### 2.3 Random effects

Following the shared parameter modeling framework, we assume that the longitudinal process {*Y*_*i*_(*t*)} and the time‐to‐event outcome *S*_*i*_ are independent conditional on the P‐spline coefficients **b**_*i*_ and all covariates. Further, we assume that **b**_*i*_ are also independent of all covariates and

(6) $$\left[\begin{array}{c} b_{i0}\\ b_{i1}\\ b_{i2}\\ \vdots \\ b_{iM} \end{array}\right]∼N\left(0,\Sigma \;=\;\left[\begin{array}{ccc} \sigma_{0}^{2} & \rho \sigma_{0}\sigma_{1} & 0\\ \rho \sigma_{0}\sigma_{1} & \sigma_{1}^{2} & 0\\ 0 & 0 & \sigma_{2}^{2}I_{M\;-\;1} \end{array}\right]\right),$$

where **I**_*M* − 1_ is the (*M* − 1)‐dimensional identity matrix. Note that the P‐spline coefficients **b**_*i*_ are used to construct time‐dependent random effects such as *W*_*r*0_(**b**_*i*_) and *W*_*r*1_(**b**_*i*_) in (3) and (4). Therefore, assuming $\left(b_{i2},\ldots ,b_{iM})∼N(0,\sigma_{2}^{2}I_{M\;-\;1}\right)$ does not mean that (*W*_10_(**b**_*i*_),…,*W*_*M*0_(**b**_*i*_)) and (*W*_11_(**b**_*i*_),…,*W*_*M*1_(**b**_*i*_)) are independent. In fact, from the functional form described in (3) and (4), it is apparent that these time‐dependent random effects are correlated with each other over time. Unlike the stationary covariance structure specified in 14, the covariance structure for time‐dependent random effects in our model is non‐stationary over time; for example, it is easy to see that $\text{cov}(W_{10}(b_{i}),W_{20}(b_{i}))\;=\;\sigma_{0}^{2}\;+\;k_{1}\rho \sigma_{0}\sigma_{1}$ but $\text{cov}(W_{20}(b_{i}),W_{30}(b_{i}))\;=\;\sigma_{0}^{2}\;+\;k_{1}k_{2}\sigma_{1}^{2}\;+\;(k_{1}\;+\;k_{2})\rho \sigma_{0}\sigma_{1}$. In sum, the serial correlation in the longitudinal process is characterized by the time‐dependent random effects with a non‐stationary covariance structure over time.

Note that $\sigma_{2}^{2}$ is the smoothing parameter that penalizes the non‐smoothness of the individual‐level P‐splines (i.e., large values of *b*_*i*2_,…,*b*_*iM*_)) and will be estimated by maximizing the marginal likelihood 6. The B‐spline approach applied in existing joint models 7 used a small number of knots (say 1–3) for each individual longitudinal trajectory but did not adapt the smoothness of the B‐splines to the data. Therefore, using high‐degree polynomial terms (e.g., cubic terms) in B‐splines could potentially lead to over‐fitting. In Section 5, we will conduct a simulation study to specifically compare the dynamic prediction performance of the joint model based on our P‐spline approach with a cubic spline approach.

The heterogeneity between individual longitudinal trajectories is determined by *σ*_0_,*σ*_1_,*σ*_2_. In the HERS example in Section 4, we parameterize *σ*_0_,*σ*_1_,*σ*_2_ using the log transformations and *ρ* using Fisher's *z*‐transform, $z(x)\;=\;\frac{1\;+\;x}{1\;-\;x}$.

## 3 Estimation

The estimation for the joint model proposed in Section 2 is based on a maximum penalized likelihood approach that maximizes the likelihood function corresponding to the joint distribution of the longitudinal and time‐to‐event outcomes $\left\{Y_{i},S_{i}^{*}\;=\;s,\delta_{i}\right\}$ times a penalty term for smoothing the population‐level P‐spline coefficients (*β*_2_,…,*β*_*M*_).

### 3.1 Likelihood

Specifically, the likelihood contribution from the *i*th patient is

(7) $$L_{i}(\theta |Y_{i},S_{i}^{*}\;=\;s,\delta_{i})\;=\;\int f(Y_{i}|b_{i};\theta )f(s,\delta_{i}|b_{i};\theta )f(b_{i};\theta )db_{i},$$

where ***θ*** denotes all unknown parameters. Let $x_{i}\;=\;{\left(x(t_{i1}),\ldots ,x(t_{in_{i}})\right)}^{T}$ and **B**_*i*_ be the *n*_*i*_ × (*M* + 1) matrix for the truncated linear basis {*B*_*l*_(*t*)} = {1,*t*,(*t* − *k*_1_)_ + _,…,(*t* − *k*_*M* − 1_)_ + _} evaluated at time points $t_{i1},\ldots ,t_{in_{i}}$. The likelihood from the longitudinal part is

$$f(Y_{i}|b_{i};\theta )\;=\;\exp\left\{-log(2\pi )n_{i}/2\;-\;log(|V_{i}|)/2\;-\;{\left(Y_{i}\;-\;\mu_{i}\right)}^{T}V_{i}^{-1}(Y_{i}\;-\;\mu_{i})/2\right\},$$

where ***μ***_*i*_ = **x**_*i*_ ***α*** + **B**_*i*_ ***β*** + **B**_*i*_ **b**_*i*_ and **V**_*i*_ is the covariance matrix obtained by evaluating the covariance function $V_{i}(t,t^{\prime })$ at time points $t_{i1},\ldots ,t_{in_{i}}$. The likelihood of the survival part is

(8) $$\begin{array}{ccc} f(s,\delta_{i}|b_{i};\theta ) & \;=\;\left\{\overset{s\;-\;1}{\underset{r\;=\;1}{\prod }}\Phi \left({\tilde{x}}_{ir}^{T}\tilde{\alpha }\;+\;\gamma_{r}^{T}W_{ir}b_{i}\right)\right\}{\left\{\Phi \left({\tilde{x}}_{is}^{T}\tilde{\alpha }\;+\;\gamma_{s}^{T}W_{is}b_{i}\right)\right\}}^{1\;-\;\delta_{i}} & \\ & \;\times {\left\{1\;-\;\Phi \left({\tilde{x}}_{is}^{T}\tilde{\alpha }\;+\;\gamma_{s}^{T}W_{is}b_{i}\right)\right\}}^{\delta_{i}}. \end{array}$$

The density *f*(**b**_*i*_;***θ***) is the multivariate normal *N*(**0**,**Σ**) defined in Section 2.3. Using the multivariate skew‐normal results 14, 18, it can be shown that the likelihood in (7) can be written analytically. Details are given in the Supporting Information.

### 3.2 Maximum penalized likelihood estimation

Finally, we incorporate the penalty term for smoothing the population‐level P‐splines coefficients $\tilde{\beta }\;=\;(\beta_{2},\ldots ,\beta_{M})$ to the likelihood function as follows:

$$PL\;=\;\overset{N}{\underset{i\;=\;1}{\prod }}L_{i}(\theta |Y_{i},S_{i}^{*}\;=\;s,\delta_{i})\;\cdot \;\exp(-\lambda {\tilde{\beta }}^{T}\tilde{\beta }),$$

where *λ*(*λ* > 0) is the smoothing parameter 6.

For a fixed value of *λ*, estimation of ***θ*** can be performed by numerical maximization of the penalized likelihood. The variance–covariance matrix of the maximum penalized likelihood estimates $\hat{\theta }$ can be estimated by the inverse of the observed Fisher information matrix. To choose an optimal value of *λ*, we first calculate the degree of freedom of population‐level P‐splines for a fixed *λ* as $df(\lambda )\;=\;\text{tr}\{{\left({\tilde{B}}^{T}\tilde{B}\;+\;2\lambda {\hat{\sigma }}_{\varepsilon }^{2}D\right)}^{-1}{\tilde{B}}^{T}\tilde{B}\}$, where $\tilde{B}\;=\;{\left(B_{1}^{T},\ldots ,B_{N}^{T}\right)}^{T},D\;=\;\left[\begin{array}{cc} 0_{2\;\times \;2} & 0_{2\;\times \;(M\;-\;1)}\\ 0_{(M\;-\;1)\;\times \;2} & I_{\left(M\;-\;1)\;\times \;(M\;-\;1\right)} \end{array}\right]$, and ${\hat{\sigma }}_{\varepsilon }^{2}$ is the maximum penalized likelihood estimate for the error variance $\sigma_{\varepsilon }^{2}$ assuming that $V_{i}(t,t^{\prime })\;=\;\sigma_{\varepsilon }^{2}I(t\;=\;t^{\prime })$ 19. Then, the Akaike information criterion (AIC) 19 is

$$AIC(\lambda )\;=\;-2log(\hat{L})\;+\;2\;\cdot \;df(\lambda )\;+\;2\;\cdot \;\dim(\tilde{\theta }),$$

where $\hat{L}\;=\;\prod_{i\;=\;1}^{N}L_{i}(\hat{\theta }|Y_{i},S_{i}^{*}\;=\;s,\delta_{i})$ is the likelihood from the joint model evaluated at the maximum penalized likelihood estimates $\hat{\theta }$ and $\tilde{\theta }$ is a subset of ***θ*** by excluding $\tilde{\beta }$. We minimize *A* *I* *C*(*λ*) to estimate the optimal *λ*.

### 3.3 Dynamic predictions of survival probabilities

In this section, we describe the procedure to perform dynamic predictions of survival probabilities based on the joint model in Section 2. Suppose that we are interested in predicting the conditional survival probability of surviving the *s*th interval given survival over the *r*th (*r* < *s*) interval for a new patient $i,\pi_{i}(s\;|\;r)\;=\;P(S_{i}\;>\;s|S_{i}\;>\;r,Y_{i}\{t(r)\},D_{N};\theta ,\lambda )$, where **Y**_*i*_{*t*(*r*)} is the series of longitudinal measurements provided by this patient up to *t*(*r*), the right end point of the *r* interval. $D_{N}\;=\;\{Y_{i},S_{i}^{*}\;=\;s,\delta_{i};i\;=\;1,\ldots ,N\}$ is the sample on which the joint model was fitted.

For a joint model with random intercept and slope, Rizopoulos 4 proposed two estimators of *π*_*i*_(*s*|*r*): (i) by evaluating *π*_*i*_(*s*|*r*) at $\hat{\theta }$ and ${\hat{b}}_{i}$, the empirical Bayes estimates of **b**_*i*_ given *S*_*i*_ > *r*,**Y**_*i*_{*t*(*r*)} and (ii) by sampling ***θ*** ^(*l*)^ from its asymptotic distribution $N(\hat{\theta },\hat{\mathrm{var}}(\hat{\theta }))$ and $b_{i}^{\left(l\right)}$ from the posterior distribution $f(b_{i}\;|\;S_{i}\;>\;r,Y_{i}\{t(r)\},\theta^{\left(l\right)})$ and then obtaining an estimate (e.g., median) from the samples of *π*_*i*_(*s*|*r*) evaluated at $\theta^{\left(l\right)},b_{i}^{\left(l\right)}$. The simulation results of 4 showed that the two estimators both performed well in terms of dynamic prediction accuracy.

In our model, the smoothing parameter *λ* is estimated separately from the other model parameters. Therefore, it seems problematic to sample from the asymptotic distribution $N(\hat{\theta },\hat{\mathrm{var}}(\hat{\theta }))$ because the population‐level P‐spline coefficients $\tilde{\beta }$ (as a subset of ***θ***) are also implicitly determined by *λ*. On the other hand, conditioning on $\hat{\theta }$ and *λ*, we can draw from $b_{i}^{\left(l\right)}∼f(b_{i}|S_{i}\;>\;r,Y_{i}\{t(r)\},\hat{\theta },\lambda )$ and calculate ${\hat{\pi }}_{i}(s\;|\;r)\;=\;\prod_{r*\;=\;r\;+\;1}^{s}\Phi \left({\tilde{x}}_{ir^{*}}^{T}\hat{\tilde{\alpha }}\;+\;\gamma_{r^{*}}^{T}W_{ir^{*}}{\hat{b}}_{i}\right)$, where ${\hat{b}}_{i}$ could be the sample mean, median, or mode of $b_{i}^{\left(l\right)}$(*l* = 1,…,*L*) and *L* is the number of Monte Carlo samples. This is the dynamic prediction procedure we will use in the analysis of the HERS data and in the simulations.

Note that $f(b_{i}\;|\;S_{i}\;>\;r,Y_{i}\{t(r)\},\hat{\theta },\lambda )$ can be shown to be the density of a multivariate skew‐normal distribution (see details in the Supporting Information). This simplifies the prediction procedure as no approximation, for example, using a Metropolis–Hastings algorithm, is needed for sampling from this distribution 4.

## 4 Application to the HIV Epidemiology Research Study data

In this section, we apply the proposed methods to the HERS data introduced in Section 1. During the follow‐up of the HERS, there were 106 HIV‐related deaths, which censored the longitudinal CD4 processes for these patients. Censoring by dropout also occurred, which was possibly related to disease progression. Previous analyses of the HERS CD4 count data 20 did not distinguish censoring by death and dropout. In our analysis, we will assume that given the random effects that characterize the individual longitudinal CD4 count process, the dropout time and the HIV‐related survival time were independent. In other words, we will focus on modeling HIV‐related survival time and treat dropout as independent censoring conditional on random effects. For those women who actually finished 12 scheduled visits, the HIV‐related survival times are treated as administratively censored.

The maximum follow‐up time was 2093 days in the HERS data, and we partition the follow‐up period into 12 intervals. Except for the first interval that is 3 months from enrollment, the remaining 11 intervals are equally spaced every 6 months, which ensures that each interval approximately contains one scheduled CD4 count measurement. We also standardize the square root of CD4 count by taking $(\sqrt{\text{CD4}}\;-\;18)/7$ to facilitate computation.

### 4.1 Models

We fit two joint models to these data. In both joint models, we assume that the covariance function of the longitudinal process is $V_{i}(t,t^{\prime })\;=\;\sigma_{\varepsilon }^{2}I(t\;=\;t^{\prime })$. We use $V_{i}(t,t^{\prime })\;=\;\sigma_{\varepsilon }^{2}I(t\;=\;t^{\prime })$ to account for measurement errors only because the specified random effects structure in (6) is used to capture the non‐stationary serial correlation over time for longitudinal data. In practice, other parametric models can be used for $V_{i}(t,t^{\prime })$, for instance, $V_{i}(t,t^{\prime })\;=\;\sigma^{2}\exp(-\varphi |t^{\prime }\;-\;t|)$, in order to characterize the remaining serial correlations.

In the first joint model (‘Model 1’), we assume that the mean function in the longitudinal CD4 count process is

$$\mu_{i}(t)\;=\;\overset{12}{\underset{l\;=\;0}{\sum }}(\beta_{l}\;+\;b_{il})B_{l}(t),$$

where *t* is the follow‐up time in days (scaled by 2093 such that *t*∈[0,1]), {*B*_*l*_(*t*)} = {1,*t*,(*t* − *k*_1_)_ + _,…,(*t* − *k*_11_)_ + _} is the truncated linear basis with knots corresponding to 3,9,…,63 months before scaling, and **b**_*i*_ = (*b*_*i*0_,…,*b*_*i*,12_) are the corresponding random P‐spline coefficients that follow the distribution specified in (6). Knot locations were chosen based on the planned visit schedule.

For comparison purposes, in the second joint model (‘Model 2’), we assume that the mean function of the longitudinal process is

$$\mu_{i}(t)\;=\;\beta_{0}\;+\;\beta_{1}t\;+\;b_{i0}\;+\;b_{i1}t,$$

and

$$\left[\begin{array}{c} b_{i0}\\ b_{i1} \end{array}\right]∼N\left(0,\left[\begin{array}{cc} \sigma_{0}^{2} & \rho \sigma_{0}\sigma_{1}\\ \rho \sigma_{0}\sigma_{1} & \sigma_{1}^{2} \end{array}\right]\right)$$

are the random intercept and time slope for the *i*th patient.

In the survival sub‐model of Model 1, based on some preliminary investigations and the findings in 17, we assume that

$$\lambda_{ir}\;=\;1\;-\;\Phi \{\alpha_{0}\;+\;\alpha_{1}\tilde{r}\;+\;\alpha_{2}{\tilde{r}}^{2}\;+\;\alpha_{3}{\text{age}}_{i}\;+\;\alpha_{4}V_{2i}\;+\;\alpha_{5}V_{3i}\;+\;\alpha_{6}V_{4i}\;+\;\gamma_{0}m_{i}(k_{r\;-\;1})\;+\;\gamma_{1}m_{i}^{\prime }(k_{r\;-\;1})\},$$

where $\tilde{r}$ is the time at the start of the *r*th time interval, age_*i*_ is the age at enrollment (standardized by taking (age_*i*_ − 35)/7) and *V*_2*i*_,*V*_3*i*_,*V*_4*i*_ are the indicator variables for HIV viral load groups (500,5000],(5000,30000],(30000,∞) at enrollment, respectively. The intercept *m*_*i*_(*k*_*r* − 1_) and slope *m* *i* *′*(*k*_*r* − 1_) of the current time interval are incorporated as time‐varying covariates. For Model 2, we assume the same survival sub‐model except that *m*(*k*_*r* − 1_) = *β*_0_ + *b*_*i*0_ + (*β*_1_ + *b*_*i*1_)*k*_*r* − 1_ and $m^{\prime }(k_{r\;-\;1})\;=\;\beta_{1}\;+\;b_{i1}$ because the time slope is assumed constant.

We use the estimation methods in Section 3 to fit the two joint models and perform dynamic predictions based on these fitted models following the procedure described in Section 3.3.

### 4.2 Summary of fitted models

**Figure 2:**
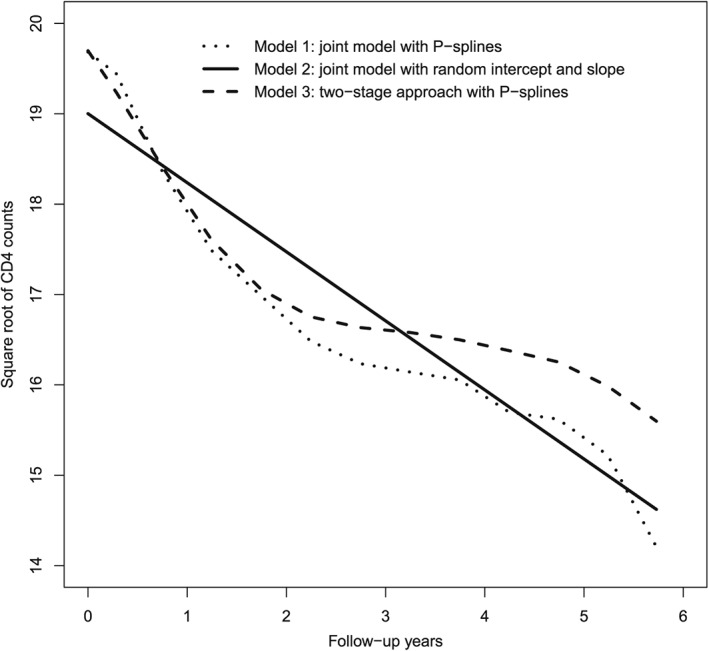
Estimated population‐level CD4 count longitudinal trajectories from the two‐stage approach and the two joint models fitted to the HIV Epidemiology Research Study data.

Figure 2 presents the estimated population‐level longitudinal CD4 count trajectories from the two joint models. Table 1 summarizes the results of survival sub‐models and variance components from Models 1 and 2. Overall, the values of AIC indicate that Model 1 provides much better fit to the observed data. The optimal value for the smoothing parameter *λ* is 0.4, which means that the effective number of parameters in the population‐level P‐splines is 9.4 (note that the number of population‐level P‐spline coefficients for penalization is 11).

**Table 1:** Parameter estimates, standard errors, and model comparison results from the two joint models fitted to the HERS data.

Parameter		Model 1	Model 2
Survival	*α*_0_	4.006 (0.342)	3.573 (0.272)
	*α*_1_	− 2.328 (0.932)	− 1.643 (0.660)
	*α*_2_	2.701 (1.028)	1.995 (0.750)
	*α*_3_	− 0.211 (0.062)	− 0.194 (0.056)
	*α*_4_	− 0.345 (0.242)	− 0.349 (0.240)
	*α*_5_	− 0.606 (0.248)	− 0.603 (0.247)
	*α*_6_	− 0.536 (0.250)	− 0.569 (0.259)
	*γ*_0_	0.775 (0.090)	0.751 (0.079)
	*γ*_1_	0.277 (0.055)	0.172 (0.058)
Others	*σ*_*ε*_	0.353 (0.004)	0.386 (0.004)
	*σ*_0_	0.916 (0.024)	0.931 (0.025)
	*σ*_1_	0.778 (0.081)	1.146 (0.043)
	*σ*_2_	0.658 (0.038)	—
	*ρ*	0.123 (0.059)	− 0.121 (0.046)
log likelihood		− 5543.876	− 5761.138
AIC		11 138.54	11 552.28
*λ*		0.4	—
df(*λ*)		9.4	—

HERS, HIV Epidemiology Research Study; AIC, Akaike information criterion.

The estimated population‐level longitudinal CD4 count trajectory from Model 1 suggests that the disease progression in the HERS cohort (decline of CD4 count) was slowing down in the middle of follow‐up when the HAART was introduced. In both models, after adjusting for enrollment age, viral load group, and the time at the start of the interval, the conditional probability of surviving each time interval was positively associated with the current intercept and time slope of the CD4 count. However, the estimates for *γ*_0_ and *γ*_1_ from Model 1 are both larger than those from Model 2, especially for *γ*_1_ (0.277 vs. 0.172) with a difference of almost two standard errors. This suggests that estimates from Model 2 are possibly attenuated because of its less flexible modeling of individual longitudinal trajectories.

In summary, the HIV‐related survival in the HERS cohort was associated with younger age and lower viral load at enrollment as well as current higher level and increasing rate of CD4 count, which is consistent with the findings in 17.

### 4.3 Dynamic predictions

In this section, we demonstrate dynamic prediction based on our flexible joint model. In particular, we exemplify it by making predictions on the conditional probabilities of surviving the next one, two, and three intervals given all baseline information and available CD4 count measurements up to the cutoff time for predictions as well as the fact that a patient was still under follow‐up at this cutoff time.

For comparison purpose, we also perform dynamic predictions using a survival model with the longitudinal outcome as a time‐varying covariate as well as a two‐stage approach. The survival model with the time‐varying covariate will follow the same structure as in the joint models, but *Y*_*ir*_, instead of estimates of *m*_*i*_(*k*_*r* − 1_) and *m* *i* *′*(*k*_*r* − 1_), is incorporated as a time‐varying covariate. The dynamic prediction procedure for this approach is similar to those used for the joint models, except that the last observed outcome *Y*_*ir*_(instead of random effect estimates) is used in the fitted survival model for prediction.

In the two‐stage approach, first we fit a linear mixed model with P‐splines to the observed longitudinal data using the same specification as for Model 1. The computation is carried out by the lme function in the R package nlme. Figure 2 also gives the estimated population‐level longitudinal CD4 count trajectory from the two‐stage approach, which overestimates the CD4 count level at later follow‐up time because it ignores the selection through survival. Using the empirical Bayes estimates of the random effects from the fitted linear mixed model, we then fit a survival model with the same specification as in Models 1 and 2. Based on the parameter point estimates from the linear mixed model, we obtain the empirical Bayes estimates of the random effects for the patients we would like to make predictions for. Finally, using these random effect estimates and the fitted survival model, we produce predicted survival probabilities over time for these patients. Note that, unlike in the joint models, the posterior distribution of the random effects used to generate empirical Bayes estimates in the two‐stage approach will not involve the observed survival data.

**Figure 3:**
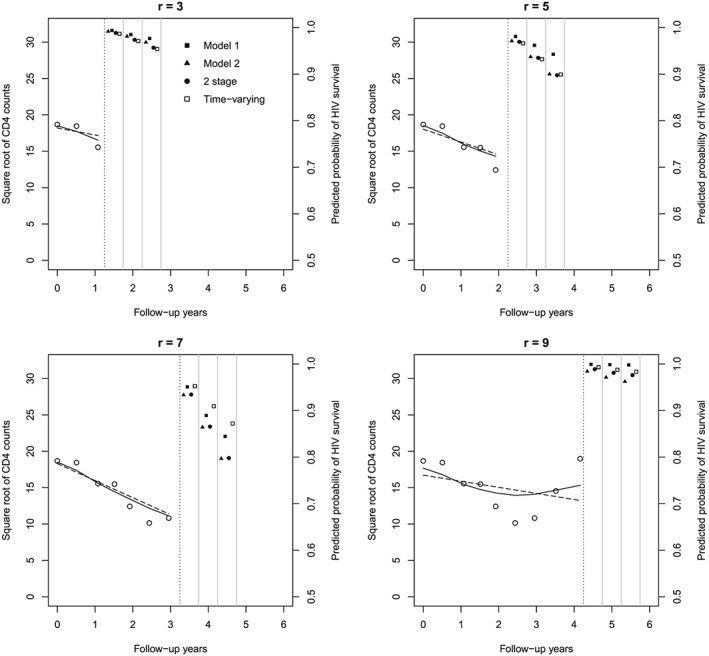
Left side of each panel: observed (standardized) square root CD4 counts and estimated individual longitudinal trajectory for patient 26 up to the prediction time r = 3,5,7,9. The dotted lines are the cutoff time for the prediction. The solid and dashed dark lines are estimated individual longitudinal trajectories (using medians of 200 samples from the posterior of **b**_i_) based on Models 1 and 2, respectively. Right side of each panel: predicted conditional probabilities of HIV survival after the next one, two, and three time intervals. Squares represent predictions from Model 1 and triangles represent predictions from Model 2 (using medians of 200 samples from the posterior of **b**_i_).

As an example, we consider patient 26 who was 37 years old with viral load (5000,30000] at enrollment. The left sides of the panels of Figure 3 present

F3 the observed (standardized) square root CD4 counts and estimated individual longitudinal trajectories for patient 26 up to the prediction time *r* = 3,5,7,9, respectively. The right sides provide predicted conditional probabilities of HIV survival after the next one, two, and three time intervals at the prediction time *r* = 3,5,7,9, respectively. For the joint models, we use medians of 200 samples from the posterior of **b**_*i*_. The same figure presented on the probit scale is given in Figure S2. At *r* = 3, the predicted conditional survival probabilities are similar for all models, while at *r* = 5,7, Model 1 predicts higher survival probability than Model 2 and the two‐stage approach. These differences could be due to different estimates of *α*_0_,…,*α*_6_ and *γ*_0_,*γ*_1_ in the survival sub‐models of Models 1 and 2. At *r* = 9, Model 1 picked up the change in CD4 counts driven by the HAART initiation and the estimated CD4 trajectory started to increase, while the estimated trajectory from Model 2 still indicated a decreasing pattern. Thus, this leads to the higher predicted survival probabilities from model 1 compared with those from Model 2 at *r* = 9, which is easier to be seen at the probit scale in Figure S2. The number of longitudinal data points required to make reliable predictions will therefore depend on the true individual trajectory, because to make predictions, we linearly extrapolate from the last observed data point before the prediction time. The survival model with the time‐varying covariate and the two‐stage approaches give similar predictions as for Model 2 for *r* = 3,5. For *r* = 9, the survival model with the time‐varying covariate gives slightly higher predicted survival probabilities, possibly because of the large value of the last observed CD4 count by the cutoff time. Overall, our joint model can capture the nonlinear change in the individual longitudinal trajectory, which is helpful to provide more accurate predictions of conditional survival probabilities.

## 5 Simulation study

In this section, we perform a simulation study to evaluate the dynamic prediction performance of the proposed flexible joint model by comparisons with (i) a survival model using the longitudinal outcome as a time‐varying covariate; (ii) a two‐stage approach that uses empirical Bayes estimates of random effects, based on a linear mixed model with P‐splines fitted to observed longitudinal data, in a subsequent survival model; (iii) a joint model with random intercept and slope only; and (iv) a joint model with cubic splines and three internal knots.

Except for the survival model with CD4 counts as time‐varying covariate, the same survival sub‐model is specified for the three joint models as well as in the two‐stage approach. We will use the ‘gold standard’ estimator of *π*_*i*_(*s*|*r*) with the true (i.e., simulated) values for random effects and true values for the parameters and evaluate the dynamic prediction performance as a function of the prediction time *r* and also the prediction window Δ*t* = *s* − *r*. The setup for the simulation is motivated by the HERS data, and details are given in Section S3.

The main conclusions drawn from the simulation study are as follows. First, the proposed joint model with P‐splines outperforms the other two joint models overall (aggregated over all prediction times). Second, the two‐stage approach performs only slightly worse than the joint model based on P‐splines in dynamic predictions, although their performances in parameter estimation are very different. The survival model using the longitudinal outcome as a time‐varying covariate performs the worst among all approaches. Third, depending on the shape of the true longitudinal trajectories and the prediction time *r*, the joint model with random intercept and slope and the joint model with cubic splines can perform similarly to the proposed joint model. Finally, the joint model with cubic splines performs the worst among the three joint models, especially at later prediction times *r*. More detailed results can be found in the Supporting Information.

Overall, our simulation results show that our flexible joint model had better dynamic prediction performance than all other approaches in comparison.

## 6 Discussion

In this paper, we developed a flexible joint model for longitudinal and time‐to‐event data with time‐dependent random effects, aiming to improve dynamic predictions by allowing for more flexible modeling of the longitudinal process. Our simulation results demonstrate that our flexible joint model with P‐splines and truncated linear basis outperformed other existing approaches in terms of parameter estimation and dynamic predictions. Moreover, in practice, it is straightforward to implement dynamic predictions based on our model because the posterior distribution of the random P‐spline coefficients is a multivariate skew‐normal distribution.

The poor performance of the joint model with cubic splines in the simulations might be explained by the fact that splines with polynomial bases tend to perform erratically beyond the boundary knot and extrapolation can be dangerous [16, Chapter 5]. For this reason, natural cubic splines are often used to add a linear constraint beyond the boundary knot. When making dynamic predictions, however, at the time of prediction, we must extrapolate an individual's trajectory beyond the current observed data for that individual. Thus, using natural cubic splines in a joint model is not very helpful if the linear constraint is only applied to the last boundary knot in the whole follow‐up. Adding further flexibility to the cubic spline model in our simulation study would be very likely to exacerbate the extrapolation problem. Our joint model with P‐splines and truncated linear basis not only offers model flexibility (compared with a joint model with random intercept and slope) but also alleviates the extrapolation problem beyond the prediction time (compared with a joint model with cubic splines). Note that this discussion is only applied to modeling individual longitudinal trajectories. For population‐level longitudinal trajectories, other spline bases with better numerical properties could be used in our model. Overall, based on the findings from our simulation study, for dynamic prediction purpose only, we do not recommend using splines with polynomial bases for modeling individual longitudinal trajectories.

Interestingly, the two‐stage approach using P‐splines and truncated linear basis also performed very well in terms of dynamic predictions, although it produced biased parameter estimates in both longitudinal and survival sub‐models. The bias–variance trade‐off is well known for prediction problems [16, Chapter 7]. The two‐stage approach and the joint model with cubic splines had similar biases in terms of parameter estimation. However, the complexity in the joint model with cubic splines also introduced more variance into the estimation, which was demonstrated by the larger variability in prediction errors for the joint model with cubic splines at later prediction times. Therefore, overall the joint model with cubic splines performed worse than the two‐stage approach for dynamic predictions in our simulations. In practice, if dynamic predictions are of main interest, two‐stage approaches can be applied without the complexity of fitting joint models. Note that the extrapolation problem for polynomial bases discussed earlier applied to the two‐stage approach as well.

Barrett *et al.* investigated the impact of discretization of the time scale on the inferences of the longitudinal and survival sub‐models 14. Their simulation studies and analysis of special cases suggested that the parameter estimates were not greatly influenced by the discretization, in particular, the covariate effects in the longitudinal and survival sub‐models. Moreover, Barrett *et al.* theoretically proved that there is no loss of information when the survival functions are linear between discrete time points 14. In practice, often there exists a natural discrete time scale, for example, dropout at pre‐specified measurement time points. For continuous‐time dropout or other continuous time to event, a discretization that ensures approximate linearity is recommended.

Using a probit model for the discretized event time, our model benefits from the straightforward implementation of dynamic prediction of survival probabilities. The probit link used in the survival sub‐model not only facilitates estimation but also naturally reflects the assumption that the discrete hazard of event occurrence depends on the *normally* distributed random effects that characterize underlying individual longitudinal trajectories. In other words, because we assume that the linear predictor in the survival sub‐model is normally distributed, it seems natural to use the probit link to transform back to the discrete hazard (probability) scale. To interpret the covariate effects in the survival sub‐model, we can present the results at the marginal survival probability scale to the subject matter experts.

Barrett *et al.* discussed the computational issues related to their joint model with time‐dependent random effect 14, which are similar in our case as the computing time is also driven primarily by calculating the multivariate normal probabilities. The R package mnormt we used applies a non‐Monte Carlo method to calculate multivariate normal probabilities up to 20 dimensions. Another R package mvtnorm uses faster quasi‐Monte Carlo methods and can accommodate dimensions up to 1000 but with less accuracy. The development in this field will certainly help improve our estimation procedure.

A limitation of our proposed model is that due to discretization of the time scale, dynamic predictions can only be made in discrete time intervals as well. But given that prediction on patient prognosis is often made in discrete time in practice, for example, 6‐year survival given that the patient is still alive at 5 years, this limitation should not be a major concern.

In our simulation study, we compared the dynamic prediction performance of three joint models, assuming that the main structure of the survival sub‐model is correctly specified. However, in practice, it is important to realize that model specification or different parameterizations in the survival sub‐model can lead to different prediction estimates for conditional survival probabilities. Rizopoulos [7, Chapter 7] compared dynamic prediction results from six joint models with different parameterizations in the survival sub‐model and found that the predicted conditional survival probabilities showed considerable variability between the six parameterizations. In practice, the choice for parameterizations in the survival sub‐model should be mainly driven by substantive knowledge. When it is not available, standard likelihood information criteria can be used to decide upon which joint model we should base the predictions 7.

## Supporting information



Supporting info item
