# Supplementary material for: Dynamic predictions using flexible joint models of longitudinal and time‐to‐event data
Source: Stat Med. 2017 Jan 22;36(9):1447–60. doi: 10.1002/sim.7209 (PMC5381717; doi:10.1002/sim.7209)
Supplement: Supplementary file 1 — Supporting info item [file SIM-36-1447-s001.zip › code_Barrett_Su_SIM-15-0679_R2.pdf]

R code for 'Dynamic Predictions using Flexible Joint Models of Longitudinal and Time-to-event Data'

Jessica Barrett
Department of Public Health and Primary Care, University of Cambridge, Strangeways Research Laboratory, Worts Causeway, Cambridge CB1 8RN, UK
email: jkb23@medschl.cam.ac.uk

and

Li Su[1]
MRC Biostatistics Unit, Robinson Way, Cambridge CB2 0SR, U.K.
email: li.su@mrc-bsu.cam.ac.uk

1 Generating a simulated dataset

The following R code generates a simulated dataset as described in Section 4.1 of the Supplementary Materials.

```
library(mnormt)

###### Functions defining the individual trajectories mi(t) ######
mxi<-function(xx, scale, fit)
# scale = vector of random effects
# fit = vector of parameters defining the population curve
{
    a=fit[1]
    b=fit[2]
    c=fit[3]
    d=fit[4]
    scale[2]*sin(a*2*pi*(xx+b))/(1+3*(xx+b)^3)+c+d*xx+(scale[1]-1)/0.3+
    xx*(scale[2]-1)/0.3
}
dmxi<-function(xx, scale, fit)
# the derivative function
{
    a=fit[1]
    b=fit[2]
    c=fit[3]
    d=fit[4]
    g=1+3*(xx+b)^3
    dg=9*(xx+b)^2
    f=scale[2]*sin((xx+b)*pi*2*a)
    df=cos((xx+b)*pi*2*a)*2*pi*scale[2]*a
    (df*g-dg*f)/g^2+d+(scale[2]-1)/0.3
}

##### Define the simulation parameters #####
Nall=850                                    # Number of individuals
ni=12                                       # Number of time intervals
knott<-(0:11)/12                            # Interval start times
nknot<- length(knott)-1                     # Number of knots
nlfit <- c(0.6343,0.7685,0.2283,-0.1924)    # Population trajectory parameters
gamma<-c(0.7,0.2)                           # Association parameters
alpha<-c(3,-2.5,2)                          # Survival parameters
```

[1] Joint first author.

```
ri<-(1:12-1)/11                              # Interval times

# Censoring probabilities
censor.prob <- c(0.9353, 0.9773, 0.9779, 0.9595, 0.9742, 0.9772, 0.9631, 0.9662,
    0.9525, 0.9682, 0.7845, 1)
# Random effects distribution parameters
means=c(1,1)
sigma=matrix(c(0.3^2, 0.3^2*(-0.2),0.3^2*(-0.2),0.3^2), ncol=2)

##### Generate a dataset ######
set.seed(20150603)
simdata<-NULL
for (i in 1:Nall)
{
    scale=rmnorm(1,means,sigma)              # Random effects
    ti=(runif(ni)+0:11)/12                   # Random observation times
    e=rnorm(ni,0,0.4)                        # Residual errors

    yi=mxi(ti,scale,nlfit)+e                 # Observed y
    slopei=dmxi(knott,scale,nlfit)           # True value of y at interval start
    inti=mxi(knott,scale,nlfit)              # True slope of y at interval start

# Survival probability for each time interval
    etai<-alpha[1]+alpha[2]*ri+alpha[3]*ri*ri+inti*gamma[1]+slopei*gamma[2]
    lambdai=pnorm(etai)

# Get survival time Si, censoring time Di, survival status by interval finalsurvi
# and censoring status by interval censori
    statusi=as.numeric(runif(ni)<lambdai)
    censori=as.numeric(runif(ni)<censor.prob)
    Si=min(which(statusi==0))
    Di=min(which(censori==0))
    if(Si==Inf){Si=13}
    if(Di==Inf){Di=13}
    finalsurvi=cumprod(statusi)
    finalcensori=cumprod(censori)

    datai<-cbind(rep(i,ni),1:12,rep(scale[1],ni),rep(scale[2],ni),ti,yi,inti,slopei,
    etai,lambdai,statusi,censori,finalsurvi,finalcensori,rep(Si,ni),rep(Di,ni))
    simdata<-rbind(simdata,datai)
}

simdata<-data.frame(simdata)
colnames(simdata)[1:4]<-c('sub','visit','scale1','scale2')
colnames(simdata)[15:16]<-c('S','D')
simfinaldata<-subset(simdata,visit<=S & visit<=D)

# Set up testing and training data
test.data<-subset(simfinaldata,sub%in%c(751:850))
train.data<-subset(simfinaldata,sub%in%c(1:750))
```

2 Fitting the models

The following R functions calculate the loglikelihood of the joint models, given a vector of model parameters.

```
library(mnormt)
library(plyr)
library(tensor)
library(numDeriv)

Sigma.is <- function(upars){
```

```
# Get variance-covariance matrix of RE distribution for random intercept/slope model
# upars = vector of variance-covariance parameters
    sig0 <- upars[1]
    sig1 <- upars[2]
    rho <- upars[3]
    mat <- matrix(c(sig0^2,rho*sig0*sig1,rho*sig0*sig1,sig1^2),nrow=2,ncol=2)
    mat
}

Sigma.spline <- function(K,upars){
# Get variance-covariance matrix of RE distribution for penalized spline model
# K = number of knots
# upars = vector of variance-covariance parameters
    sig0 <- upars[1]
    sig1 <- upars[2]
    sig2 <- upars[3]
    rho <- upars[4]
    mat <- matrix(0,nrow=K+2,ncol=K+2)
    mat[1:2,1:2] <- c(sig0^2,rho*sig0*sig1,rho*sig0*sig1,sig1^2)
    mat[-(1:2),-(1:2)] <- diag(K)*sig2^2
    mat
}

Sigma.bs <- function(upars){
# Get variance-covariance matrix of RE distribution for cubic spline model
# upars = vector of variance-covariance parameters
    nu <- length(upars)
    mat <- matrix(0,nrow=nu, ncol=nu)
    diag(mat) <- upars^2
    mat
}

L.is <- function(p=nt,gampars, tbreaks){
# Get L-matrix multiplying REs in survival model for random intercept/slope model
# p = number of time intervals
# gampars = vector of association parameters
# tbreaks = vector of interval start times
    gam0 <- gampars[1]
    gam1 <- gampars[2]
    mat <- matrix(0,nrow=p,ncol=2)
    mat[,1] <- gam0
    mat[,2] <- gam0*tbreaks + gam1
    mat
}

L.spline <- function(knts, nt, gampars){
# Get L-matrix multiplying REs in survival model for penalized spline model
# knts = vector of knot locations
# nt = number of time intervals
# gampars = vector of association parameters
    K <- length(knts)
    gam1 <- gampars[1]
    gam2 <- gampars[2]
    mat <- matrix(0,nrow=nt,ncol=(K+2))
    mat[,1] <- mat[,1] + gam1
    temp <- outer(c(0,knts),c(0,knts),"-")
    temp1 <- 1*(temp>=0)         # matrix with ones on diagonal and lower triangle
    temp <- temp*(temp>0)
    mat[,-1] <- gam1*temp + gam2*temp1
    mat
}

L.bs <- function(gampars, tbreaks, knts, nu, nt){
# Get L-matrix multiplying REs in survival model for cubic spline model
# gampars = vector of association parameters
# tbreaks = vector of interval start times
# knts = vector of knot locations
# nu = number of random effects
```

```
# nt = number of time intervals
    gam1 <- gampars[1]
    gam2 <- gampars[2]
# spline time differences
    Z <- outer(tbreaks,knts,'-')
    Z <- Z*(Z>0)
# temp1 contains gam1 coefficients
    temp1 <- matrix(0,nrow=nt,ncol=nu)
    temp1[,1] <- 1
    temp1[,2] <- tbreaks
    temp1[,3] <- tbreaks^2
    temp1[,4] <- tbreaks^3
    temp1[,-(1:4)] <- Z^3
# temp2 contains gam2 coefficients
    temp2 <- matrix(0,nrow=nt,ncol=nu)
    temp2[,2] <- 1
    temp2[,3] <- 2*tbreaks
    temp2[,4] <- 3*tbreaks^2
    temp2[,-(1:4)] <- 3*Z^2
# add gam1 terms and gam2 terms for L matrix
    mat <- gam1*temp1 + gam2*temp2
    mat
}

frhoinv <- function(x){
# invert the rho transformation
    x <- exp(x)/(1+exp(x))
    2*x-1
}

simloglik <- function(pars, ses=NULL, dat, surv, quad, tbreaks, pmnormtol=1e-3,
    knts, lamda, logsd.csplines, printpars=F, model){
# Calculates the loglikelihood (see section 1 of Supplementary Materials)
# pars = vector of model parameters in the order:
#       y model fixed effects
#       t model fixed effects
#       y model residual variance
#       RE distribution variance parameters
#       t model association parameters
# all variance parameters are defined on the log-scale
# and correlations are transformed to the real line
# all parameters are scaled by the vector ses
# dat = dataframe of longitudinal data: id, y, visittime
# surv = dataframe of survival data (one row per subject): id, status (=0 event/1 censoring), time
# quad = TRUE to include quadratic time in survival model
# tbreaks = vector of interval start times
# pmnormtol = tolerance for multivariate cumulative normal distribution
# knts = vector of knot locations
# lamda = smoothing parameter
# logsd.csplines = fixed variances of REs for cubic spline model
# printpars = TRUE to print model parameters
# model is one of "intslope" (intercept/slope), "spline" (penalized linear spline),
# "Cspline" (cubic spline)

# Extract parameters
    if(!is.null(ses))   pars <- pars*ses
    if(printpars) print(pars)
    n <- dim(surv)[1]
    ny <- dim(dat)[1]
    nt <- max(surv$time)
    K <- length(knts)

    py <- ncol(dat)+K-1
    if(model=="Cspline") py <- py+2
    if(quad){ pt <- ncol(surv) } else {  pt <- ncol(surv)-1 }
    pyt <- py+pt
    by <- pars[1:py]
    bt <- pars[(py+1):pyt]
```

```
    sig.y <- exp(pars[(pyt+1)])

# Get RE variance-covariance matrix and L matrix
    if(model=="spline"){
        rho <- 2*exp(pars[(pyt+5)])/(1+exp(pars[(pyt+5)]))-1
        upars <- c(exp(pars[(pyt+2):(pyt+4)]),rho)
        gampars <- pars[(pyt+6):(pyt+7)]
        Sig.u <- Sigma.spline(K=K, upars)
        L <- L.spline(knts=knts, nt=nt, gampars)
        nu <- K+2
    }
    if(model=="intslope"){
        rho <- 2*exp(pars[pyt+4])/(1+exp(pars[pyt+4]))-1
        upars <- c(exp(pars[(pyt+2):(pyt+3)]),rho)
        gampars <- pars[(pyt+5):(pyt+6)]
        Sig.u <- Sigma.is(upars)
        L <- L.is(p=nt, gampars, tbreaks=tbreaks)
        nu <- 2
    }
    if(model=="Cspline"){
        upars <- c(exp(pars[(pyt+2):(pyt+4)]),exp(logsd.csplines))
        gampars <- pars[(pyt+5):(pyt+6)]
        Sig.u <- Sigma.bs(upars)
        L <- L.bs(gampars=gampars, tbreaks=tbreaks, knts=knts, nu=length(upars), nt=nt)
        nu <- length(upars)
    }

# Extract data
    id <- dat$id
    y <- dat$y
    time.y <- dat$visittime
    status <- surv$status
    time <- surv$time

# Get longitudinal X matrix and Z matrix
    X.y <- dat[,-(1:3)]
    if(model=="intslope"){
        X.y <- cbind(1,time.y,X.y)
    }
    if(model=="spline"){
        Z <- outer(time.y,knts,"-")
        Z <- Z*(Z>0)
        X.y <- cbind(1,time.y,X.y,Z)
    }
    if(model=="Cspline"){
        Z <- outer(time.y,knts,"-")
        Z <- Z^3*(Z>0)
        X.y <- cbind(1,time.y,time.y^2,time.y^3,Z,X.y)
    }

# Get survival X matrix
    X.t <- surv[,-(1:3)]
    intervaltime <- rep(tbreaks, n)
    ind <- rep(1:n,rep(nt,n))
    if(quad){
        X.t <- cbind(1,intervaltime,intervaltime^2,as.matrix(X.t)[ind,])
    } else {
        X.t <- cbind(1,intervaltime,as.matrix(X.t)[ind,])
    }

# Add population y intercept and population y slope to survival X matrix
# These will be multiplied by gammas to give association with current y value and slope
    if(model=="intslope"){
        X.t <- cbind(X.t,by[1]+by[2]*intervaltime,by[2])
    }
    if(model=="spline"){
        popslope <- cumsum(by[-1])
        Zt <- outer(tbreaks,tbreaks,"-")
```

```
        popintercept <- cbind(1,(Zt * (Zt>0))) %*% by
        popslope <- rep(popslope,n)
        popintercept <- rep(popintercept,n)
        X.t <- cbind(X.t, popintercept, popslope)
    }
    if(model=="Cspline"){
        Zt <- outer(tbreaks,knts,"-")
        Zt <- Zt*(Zt>0)
        popintercept <- cbind(1, tbreaks, tbreaks^2, tbreaks^3, Zt^3) %*% by[1:(K+4)]
        popintercept <- rep(popintercept,n)
        popslope <- cbind(0, 1, 2*tbreaks, 3*tbreaks^2, 3*Zt^2) %*% by[1:(K+4)]
        popslope <- rep(popslope,n)
        X.t <- cbind(X.t, popintercept, popslope)
    }

# Get inverse H matrix
    if(model=="intslope"){
        BinvVB <- array(0,dim=c(2,2,n))
        BinvVB[1,1,] <- table(factor(id,levels=surv$id))
        BinvVB[1,2,] <- tapply(time.y,factor(id,levels=surv$id),sum)
        BinvVB[2,1,] <- BinvVB[1,2,]
        BinvVB[2,2,] <- tapply(time.y^2,factor(id,levels=surv$id),sum)
        BinvVB <- ifelse(is.na(BinvVB),0,BinvVB)
        BinvVB <- BinvVB / sig.y^2
    }
    if(model=="spline"|model=="Cspline"){
        BinvVB <- array(0,dim=c(nu, nu, n))
        if(model=="spline")  tmp <- cbind(1,time.y,Z)
        if(model=="Cspline")  tmp <- cbind(1,time.y,time.y^2,time.y^3,Z)
        for(i in 1:nu){
            for(j in 1:nu){
                BB <- tmp[,i]*tmp[,j]
                BinvVB[i,j,] <- tapply(BB,factor(id,levels=surv$id),sum)
            }
        }
        BinvVB <- ifelse(is.na(BinvVB),0,BinvVB)
        BinvVB <- BinvVB / sig.y^2
    }
    Sig.u.inv <- solve(Sig.u)
    getinvH <- function(M){chol2inv(chol(M+Sig.u.inv))}
    Hinv <- aaply(BinvVB,3,getinvH)
    Hinv <- aperm(Hinv,perm=c(2,3,1))

# Get h vector
    yran <- y - as.matrix(X.y) %*% by
    if(model=="spline"|model=="Cspline"){
        BinVyran <- matrix(nrow=n,ncol=nu)
        for(i in 1:nu){
            By <- tmp[,i]*yran
            BinVyran[,i] <- tapply(By,factor(id,levels=surv$id),sum)/sig.y^2
        }
    }
    if(model=="intslope"){
        BinVyran <- matrix(nrow=n,ncol=2)
        BinVyran[,1] <- tapply(yran,factor(id,levels=surv$id),sum) /sig.y^2
        BinVyran[,2] <- tapply(yran*time.y,factor(id,levels=surv$id),sum) /sig.y^2
        BinVyran <- ifelse(is.na(BinVyran),0,BinVyran)
    }
    h <- matrix(nrow=nu,ncol=n)
    for(j in 1:nu){
            tmp <- Hinv[j,,] * t(BinVyran)
            h[j,] <- apply(tmp,2,sum)
    }

# Get argument of cumulative normal distribution
    betaX <- as.matrix(X.t) %*% as.matrix(c(bt,gampars))
    betaX <- matrix(betaX, nrow=nt, ncol=n)
    q.Phi <- betaX + L%*%h
```

```
# Calculate cumulative normal probabilities of arguments q.Phi for multivariate normal distribution
# with mean = 0 and variance matrix = I+L Hinv Ltranspose
    Phi <- matrix(0,nrow=n,ncol=(nt+1))
    Phi[,1] <- 1
    I <- array(diag(nt),dim=c(nt,nt,n))
    for(i in 1:nt){
        Inew <- I[1:i,1:i,]
        Lnew <- L[1:i,]
        if(i==1){
            tmp <- tensor(as.matrix(Lnew),Hinv,1,1)
            LHL <- tensor(tmp,as.matrix(Lnew),2,1)
        } else {
            tmp <- tensor(Lnew,Hinv,2,1)
            LHL <- tensor(Lnew,tmp,2,2)
        }

            Sig.Phi <- Inew + LHL
            mu.Phi <- rep(0,i)

            ids1 <- c(which((time==i)&status==0),which((time==(i+1))&status==0))
            ids2 <- which((time==i)&status==1)
            ids <- c(ids1,ids2)

            p.Phi <- rep(0,n)
            if(i==1){
            p.Phi[ids] <- pnorm(q.Phi[1,ids],mean=0,sd=sqrt(Sig.Phi[ids]))
            } else {
            for(k in ids){
                    p.Phi[k] <- pmnorm(q.Phi[1:i,k],mu.Phi,Sig.Phi[,,k],abseps=pmnormtol)
            }
            }
            Phi[,(i+1)] <- p.Phi
    }

# Matrix Psi contains differences in cumulative normal probabilities
    Psi <- matrix(nrow=n,ncol=nt)
    for(i in 1:nt){
            Psi[,i] <- Phi[,i] - Phi[,(i+1)]
    }

# Get all the terms contributing to the loglikelihood
    term1 <- -ny*log(sig.y)
    term2 <- - 0.5*n*log(det(Sig.u))
    term3 <- 0.5*sum(log(apply(Hinv,3,det)))
    term4 <- - sum(yran^2)/(2*sig.y^2)
    tmp <- matrix(nrow=nu,ncol=nu)
    for(i in 1:nu){
            for(j in 1:nu){
            tmp[i,j] <- sum(h[,i] * Hinv[i,j,] * h[,j])
            }
    }
    term5 <- 0.5 * sum(tmp)
# contribution from those who experience events
    term6 <- 0
    for(i in 1:nt){
            ids <- which((time==i)&status==0)
            term6 <- term6+ sum(log(Psi[ids,i]))
    }
# contribution from censored individuals
    term7 <- 0
    for(i in 1:nt){
            ids <- which((time==i)&status==1)
            term7 <- term7 + sum(log(Phi[ids,(i+1)]))
    }

    loglik <- term1 + term2 + term3 + term4 + term5 + term6 + term7
```

```
# Add penalty term for penalized spline model
    if(model == "spline"){
        term8 <- -lamda*sum(by[(py-K+1):py]^2) + K*log(lamda)/2
        loglik <- loglik + term8
    }

    if (is.finite(loglik)) {
        return(-loglik)
        } else {
            return(1e+09)
        }
}
```

The following R functions find the parameter estimates by maximizing the loglikelihood.

```
# Functions to transform correlation parameter to the real line
frho <- function(p){
    p <- (p+1)/2
    log(p/(1-p))
}
frhodiff <- function(p){
    p <- 2/(1-p^2)
}

fitsimmodel <- function(Dat=dat.y, Survdat=dat.t, tbreaks=timebreaks, knts=timebreaks[-1], lamda=NA,
    logsd.csplines=NA, printpars=F, maxiter=10000, pmnormtol=1e-3, model="spline"){
# Fits the joint models
# Dat = dataframe of longitudinal data: id, y, visittime
# Survdat = dataframe of survival data (one row per subject): id, status (=0 event/1 censoring), time
# tbreaks = vector of interval start times
# knts = vector of knot locations
# lamda = fixed value of smoothing parameter
# logsd.csplines = fixed variances of REs for cubic spline model
# printpars = TRUE to print model parameters at each iteration
# maxiter = maximum number of iterations for optimization routine
# pmnormtol = tolerance for multivariate cumulative normal distribution
# model = one of "intslope" (intercept/slope), "spline" (penalized linear spline),
# "Cspline" (cubic spline)

# Use initial estimates and standard errors from two-stage simulations
# initial estimates and standard errors for longitudinal model
    if(model=="intslope"){
        meanpars.y <- c(0.0539, -0.0070)
        varpars <- c(-0.9020, 0.0098, 0.0560, -0.2565)
        se.y <- c(0.0356, 0.0394)
        se.var <- c(0.0086, 0.0256, 0.0276, 0.0343)
        se.var[4] <- frhodiff(varpars[4])*se.var[4]
        varpars[4] <- frho(varpars[4])
        knts <- NULL
    }
    if(model=="spline"){
        meanpars.y <- c(0.2472, -1.4176, 0.3224, 0.5220, 0.3853, 0.2797, 0.1367, 0.0710, -0.0180,
            -0.0663, -0.0840, -0.0676, -0.1056)
        varpars <- c(-0.9203, 0.0065, -0.0646, -1.4520, -0.2492)
        se.y <- c(0.0366, 0.2250, 0.2113, 0.1672, 0.1527, 0.1406, 0.1406, 0.1452, 0.1508, 0.1444, 0.1607, [illegible]
        se.var <- c(0.0082, 0.0257, 0.0490, 0.1756, 0.0398)
        se.var[5] <- frhodiff(varpars[5])*se.var[5]
        varpars[5] <- frho(varpars[5])
    }
    if(model=="Cspline"){
        meanpars.y <- c(0.2704, -2.0980, 4.7836, -3.4076, 0.7276, 1.7077, 0.4934)
        varpars <- c(-0.9173, -0.0164, -0.1032, -0.6883)
        se.y <- c(0.0401, 0.5606, 3.2544, 5.3929, 7.1684, 3.9896, 7.6671)
        se.var <- c(0.0085, 0.0254, 0.0437, 0.1158)
    }
# initial estimates and standard errors for survival model
    meanpars.t <- c(3.0107, -2.4895, 1.9739)
```

```
    gampars <- c(0.7063, 0.2006)
    se.t <- c(0.1505, 0.5922, 0.5896)
    se.gam <- c(0.0585, 0.0428)
# inits = vector of initial estimates
    inits <- c(meanpars.y,meanpars.t,varpars,gampars)
# initses = vector of initial standard errors.
    initses <- c(se.y,se.t,se.var,se.gam)
    initses <- ifelse(initses<0.01,0.01,initses)
# Rescale all parameters by initial standard errors to improve convergence of optimisation routine
    inits <- inits/initses

# fit the model
    fit <- nlminb(start=inits, objective=simloglik, dat=Dat, surv=Survdat, ses=initses, model=model,
            knts=knts, lamda=lamda, logsd.csplines=logsd.csplines, quad=T, tbreaks=tbreaks,
            printpars=printpars, pmnormtol=pmnormtol, control=list(iter.max=maxiter, eval.max=maxiter))
    estimate <- fit$par*initses
    fithess <- hessian(func=simloglik, x=fit$par, dat=Dat, surv=Survdat, ses=initses, model=model,
            knts=knts, lamda=lamda, logsd.csplines=logsd.csplines, quad=T, tbreaks=tbreaks, printpars=F,
            pmnormtol=pmnormtol)
    fithess <- outer(1/initses,1/initses)*fithess
    rania <- solve(fithess)
    fitses <- sqrt(diag(rania))

# Fix names of model results
    longnames <- c("Intercept",names(Dat)[-(1:2)])
    if(model=="spline"){
        longnames <- c(longnames, paste("B",(3:(length(knts)+2)), sep=""))
    }
    if(model=="Cspline"){
        longnames <- c("Intercept","Time","Time^2","Time^3", paste("B",(5:(length(knts)+4)), sep=""),
                    names(Dat)[-(1:3)])
    }
    survnames <- c("Intercept","Time","Time^2",names(Survdat)[-(1:3)])
    if(model=="intslope"){
        names(estimate) <- c(longnames,survnames,"sigmay","sigma0","sigma1","rho","gamma0","gamma1")
    }
    if(model=="spline"){
        names(estimate) <- c(longnames,survnames,"sigmay","sigma0","sigma1","sigmaB","rho","gamma0",
                        "gamma1")
    }
    if(model=="Cspline"){
        names(estimate) <- c(longnames,survnames,"sigmay", paste("sigmaB",(1:(length(knts))), sep=""),
                        "gamma0","gamma1")
    }
    names(fitses) <- names(estimate)

    out <- list(estimate=estimate, se=fitses, fit=fit, fithess=fithess, lamda=lamda,
            logsd.csplines=logsd.csplines, model=model, method=method)
    out
}
```

The following R functions print the results for the joint models.

```
frhoinv <- function(x){
    x <- exp(x)/(1+exp(x))
    2*x-1
}

frhoinvdiff <- function(x){
    2*exp(x)/(1+exp(x))^2
}

summary.modelfit <- function(modelfit, digits=3){
    model <- modelfit$model
    est <- modelfit$estimate
    se <- modelfit$se
```

```
# Print which model has been fitted.
    if(model=="intslope") cat("Random intercept/slope model.\n")
    if(model=="spline")  cat("Penalised spline model with random spline coefficients.\n")
    if(model=="Cspline")  cat("Cubic spline model with random spline coefficients.\n")

# Print convergence information
    if(modelfit$fit$convergence == 0){ cat("\nOptimisation routine converged.\n\n")
    } else { cat("\n Warning: optimisation routine didn't converge (", modelfit$fit$message,")\n\n") }

# Identify variance and correlation parameters
    ind.sigy <- which(names(est)=="sigmay")
    ind.sig1 <- which(names(est)=="sigma1")
    ind.rho <- which(names(est)=="rho")
    ind.gam0 <- which(names(est)=="gamma0")
    ind.gam1 <- which(names(est)=="gamma1")
    if(model=="spline"){
        ind.sigB <- which(names(est)=="sigmaB")
    }

# Transform variance and correlation parameters back to their original scales
    if(model=="intslope"){
        estnew <- c(est[1:(ind.sigy-1)],exp(est[ind.sigy:ind.sig1]),frhoinv(est[ind.rho]),
                est[ind.gam0:ind.gam1])
        senew <- c(se[1:(ind.sigy-1)],exp(est[ind.sigy:ind.sig1])*se[ind.sigy:ind.sig1],
                frhoinvdiff(est[ind.rho])*se[ind.rho],se[ind.gam0:ind.gam1])
    }
    if(model=="spline"){
        estnew <- c(est[1:(ind.sigy-1)],exp(est[ind.sigy:ind.sigB]),frhoinv(est[ind.rho]),
                est[ind.gam0:ind.gam1])
        senew <- c(se[1:(ind.sigy-1)],exp(est[ind.sigy:ind.sigB])*se[ind.sigy:ind.sigB],
                frhoinvdiff(est[ind.rho])*se[ind.rho],se[ind.gam0:ind.gam1])
    }
    if(model=="Cspline"){
        estnew <- c(est[1:(ind.sigy-1)],exp(est[ind.sigy:(ind.gam0-1)]),est[ind.gam0:ind.gam1])
        senew <- c(se[1:(ind.sigy-1)],exp(est[ind.sigy:(ind.gam0-1)])*se[ind.sigy:(ind.gam0-1)],
                se[ind.gam0:ind.gam1])
    }

# Print results
    cat("Estimates \n")
    print(round(estnew,dig=digits))
    cat("\n Standard errors \n")
    print(round(senew,dig=digits))
    if(model=="spline")  cat("\n\nFixed smoothing parameter, lamda = ", modelfit$lamda,"\n")
    if(model=="Cspline")  cat("\n\nFixed standard deviations of cubic spline random effects are:\n",
                        exp(modelfit$logsd.csplines),"\n")
}
```

The following R code fits the survival model with the time-varying covariate, the two-stage model and the joint models.

```
library(nlme)

#####   Set up the data   #####
dat.y <- with(train.data,data.frame(sub,yi,ti))
names(dat.y) <- c("id", "y", "visittime")
id <- tapply(train.data$sub,train.data$sub,mean)
status <- tapply(train.data$finalsurvi,train.data$sub,min)
time <- tapply(train.data$visit,train.data$sub,max)
dat.t <- data.frame(id,status,time)
timebreaks <- (0:11)/12
knott<-(0:11)/12
nknot<-length(knott)-1
T<-train.data$visit
r<-(T-1)/11
```

```
#####   Fit the model with a time-varying covariate   #####
train.data$r <-(train.data$visit -1)/11
success.timev <-try(surv.fit.timev <-glm(finalsurvi~r+I(r^2)+yi,family=binomial(link='probit'),
                data=train.data))
summary(success.timev)

#####   Fit the two-stage model   #####
# fit the longitudinal part
Z <-outer(train.data$ti,knott[-1],'-')
Z <-Z*(Z>0)
Id<-factor(rep(1,nrow(train.data)))
Z.subject<-outer(train.data$ti,knott[-1],'-')
Z.subject<-Z.subject*(Z.subject>0)
fit.spline<-lme(yi~ti,random=list(Id=pdIdent(~Z-1),sub=pdSymm(~ti),sub=pdIdent(~Z.subject-1)),
            data=train.data)

# Fixed effects from longitudinal model
re.spline<-random.effects(fit.spline)
beta<-as.matrix(unlist(c(fit.spline$coeff$fixed,re.spline$Id)))
# Random effects from longitudinal model
res <- cbind(re.spline[[2]],re.spline[[3]])
# Add random and fixed effects to give coefficients of spline basis in longitudinal model
Beta <- sweep(res,2,beta,FUN="+")
Beta <- Beta[train.data$sub,]

# fit the survival part
# Create Z matrices for contribution from B-spline coefficients to intercept and slope at each interval
# (see equations (3) and (4) of main paper)
Zslope<-Zint<-matrix(0, nrow(train.data), nknot+1)
for (l in 1:(nknot+1)) {
    Zslope[,l]<-as.numeric(train.data$visit>=l)
}
Zint[,1]<-1
for (l in 2:(nknot+1)){
    Zint[,l]<-as.numeric(train.data$visit>=l)*(knott[train.data$visit]-knott[l-1])
}
# Get the intercepts and slopes at the start of each interval
intercept <- Beta[,-13]*Zint
intercept <- apply(intercept,1,sum)
slope <-  Beta[,-1]*Zslope
slope <- apply(slope,1,sum)
# Survival model
surv.spline<-glm(finalsurvi~r+I(r^2)+intercept+slope,family=binomial(link='probit'),data=train.data)

#####    Fit the intercept/slope model   #####
simfit.IS <- fitsimmodel(dat.y, dat.t, timebreaks, model="intslope")
summary.modelfit(simfit.IS)

#####    Fit the penalised spline model, estimate lamda from complete data longitudinal model fit
#####
simfit.PS <- fitsimmodel(dat.y, dat.t, timebreaks, knts=timebreaks[-1], lamda=3.232, model="spline")
summary.modelfit(simfit.PS)

#####    Fit the cubic spline model   #####
simfit.CS <- fitsimmodel(dat.y, dat.t, timebreaks, knts=c(0.25,0.5,0.75),
        logsd.csplines=c(-8.3456,-8.0873,-6.9717,-3.5608), model="Cspline")
summary.modelfit(simfit.CS)
```

3 Dynamic predictions

The following R function calculates the probability of one subject surviving another ΔT time intervals for the time-varying covariate model.

```
predict.all.t.sim.timev <- function(yi,newsXi,Ti,beta,gamma,deltaTi){
# yi = longitudinal data up to current time interval for subject i
# newsXi = survival model X matrix up to and including prediction time interval(s)
# Ti = time intervals up to current time interval
# beta = survival model coefficients
# gamma = coefficient of time-varying covariate
# deltaTi = number of time intervals to predict survival for
    nt <- max(Ti)
    yilast<-yi[length(yi)]
    qq<- newsXi%*%beta+rep(yilast,(nt+deltaTi))*gamma
    ppj<-prod(pnorm(qq[(nt+1):(nt+deltaTi)],lower.tail=T))
    out <- c(qnorm(ppj), ppj)  ## predicted survival probabilities
    return(out)
}
```

The following R function calculates the probability of one subject surviving another ΔT time intervals for the two-stage model.

```
 library(modeest)

modeb<-function(mySamples){
# get the mode
  mlv(mySamples, method = "kernel")$M
}

predict.all.t.sim.2stage <- function(yi,yXi,yZi,newsXi,sXi,Ti,theta,beta,gamma,Lmat,Lpredmat,Lpopmat,
Lpoppredmat,ySigma,reSigma,deltaTi,nMC=200){
# yi = longitudinal data up to current time interval for subject i
# yXi = longitudinal model X matrix up to current time interval
# yZi = longitudinal model RE matrix up to current time interval
# newsXi = survival model X matrix up to and including prediction time interval(s)
# sXi = survival model X matrix up to current time interval
# Ti = time intervals up to current time interval
# theta = longitudinal model coefficients
# beta = survival model coefficients
# gamma = association parameter coefficients
# Lmat = linear combinations of random effects for survival model up to current time interval
#       (intercept L matrix cbinded with slope L matrix)
# Lpredmat = linear combinations of random effects for survival model up to and including prediction
#       time interval(s) (intercept L matrix cbinded with slope L matrix)
# Lpopmat = linear combinations of longitudinal fixed effects for survival model up to current
#       time interval (intercept L matrix cbinded with slope L matrix)
# Lpoppredmat =  linear combinations of longitudinal fixed effects for survival model up to and
#       including prediction time interval(s) (intercept L matrix cbinded with slope L matrix)
# ySigma = longitudinal model residual variance
# reSigma = variance matrix of RE distribution
# deltaTi = number of time intervals to predict survival for
# nMC = number of Monte Carlo iterations

    nt <- max(Ti)

# Random effects distribution conditional on past y observations
# E[u|Y] = h, V[u|Y] = H^{-1}

# Get conditional variance matrix
    sig.y <- sqrt(ySigma)
    Sig.u <-reSigma  #random effect variance matrix
    BinvVB <- t(yZi) %*% yZi / sig.y^2
    Sig.u.inv <- solve(Sig.u)
    Hinv <- chol2inv(chol(BinvVB+Sig.u.inv))
    Sigcond <- Hinv

# Get conditional mean
    yran <- yi - yXi%*%theta
    BinVyran <- sweep(yZi,1,yran,"*")
```

```
    BinVyran <- apply(BinVyran,2,sum) / sig.y^2
    h <- Hinv%*%BinVyran
    mucond <-  h

# Draw nMC samples from conditional RE distribution and get mean, median and mode
    uall<-NULL
    for(l in 1:nMC){
        u <- rmnorm(n=1, mean=mucond, varcov=Sigcond)
        uall<-rbind(uall,u)
    }
    ustat1=apply(uall,2,mean)
    ustat2=apply(uall,2,median)
    ustat3=apply(uall,2, modeb)

# L and Lpop matrices define linear combinations of random and fixed effects for survival model
    renum<-dim(reSigma)[2]
    fixnum<-length(theta)
    L=Lpred=0
    Lpop=Lpoppred=0
    for(j in 1:length(gamma))
    {
        L=L+Lmat[,((j-1)*renum+1):(j*renum)]*gamma[j]
        Lpred=Lpred+Lpredmat[,((j-1)*renum+1):(j*renum)]*gamma[j]
        Lpop=Lpop+Lpopmat[,((j-1)*fixnum+1):(j*fixnum)]*gamma[j]
        Lpoppred=Lpoppred+Lpoppredmat[,((j-1)*fixnum+1):(j*fixnum)]*gamma[j]
    }
    if(class(L)=='numeric'){L=t(as.matrix(L))}
    if(class(Lpop)=='numeric'){Lpop=t(as.matrix(Lpop))}

# Linear predictors for surviving each time interval
    qq1<- newsXi%*%beta+Lpred%*%(ustat1)+Lpoppred%*%theta
    qq2<- newsXi%*%beta+Lpred%*%(ustat2)+Lpoppred%*%theta
    qq3<- newsXi%*%beta+Lpred%*%(ustat3)+Lpoppred%*%theta

# Predicted survival probabilities
    ppj1<-prod(pnorm(qq1[(nt+1):(nt+deltaTi)],lower.tail=T))
    ppj2<-prod(pnorm(qq2[(nt+1):(nt+deltaTi)],lower.tail=T))
    ppj3<-prod(pnorm(qq3[(nt+1):(nt+deltaTi)],lower.tail=T))

    out <- c(qnorm(ppj1), ppj1,qnorm(ppj2), ppj2,qnorm(ppj3), ppj3 )
    return(out)
}
```

The following R functions calculate the probability of one subject surviving another ΔT time intervals for the joint models.

```
## Functions
Sigma.spline2 <- function(K,upars){
  sig0 <- upars[1]
  sig1 <- upars[2]
  sig2 <- upars[3]
  rho <- upars[4]
  mat <- matrix(0,nrow=K+2,ncol=K+2)
  mat[1:2,1:2] <- c(sig0,rho*sqrt(sig0)*sqrt(sig1),rho*sqrt(sig0)*sqrt(sig1),sig1)
  mat[-(1:2),-(1:2)] <- diag(K)*sig2
  mat
}

Sigma.is2 <- function(upars){
  sig0 <- upars[1]
  sig1 <- upars[2]
  rho <- upars[3]
  mat <- matrix(c(sig0,rho*sqrt(sig0)*sqrt(sig1),rho*sqrt(sig0)*sqrt(sig1),sig1),nrow=2,ncol=2)
  mat
}
```

```
predict.all.t.sim <- function(yi,yXi,yZi,newsXi,sXi,Ti,theta,beta,gamma,Lmat,Lpredmat,Lpopmat,
Lpoppredmat,ySigma,reSigma,deltaTi,nMC=200,jmax=100)
{
# yi = longitudinal data up to current time interval for subject i
# yXi = longitudinal model X matrix up to current time interval
# yZi = longitudinal model RE matrix up to current time interval
# newsXi = survival model X matrix up to and including prediction time interval(s)
# sXi = survival model X matrix up to current time interval
# Ti = time intervals up to current time interval
# theta = longitudinal model coefficients
# beta = survival model coefficients
# gamma = association parameter coefficients
# Lmat = linear combinations of random effects for survival model up to current time interval
#       (intercept L matrix cbinded with slope L matrix)
# Lpredmat = linear combinations of random effects for survival model up to and including prediction
#       time interval(s) (intercept L matrix cbinded with slope L matrix)
# Lpopmat = linear combinations of longitudinal fixed effects for survival model up to current
#       time interval (intercept L matrix cbinded with slope L matrix)
# Lpoppredmat =  linear combinations of longitudinal fixed effects for survival model up to and
#       including prediction time interval(s) (intercept L matrix cbinded with slope L matrix)
# ySigma = longitudinal model residual variance
# reSigma = variance matrix of RE distribution
# deltaTi = number of time intervals to predict survival for
# nMC = number of Monte Carlo iterations

    nt <- max(Ti)

# Posterior distribution of random effects is multivariate skew-normal
# (see section 2 of Supplementary materials)

# Get inverse H matrix
    sig.y <- sqrt(ySigma)  # error variance
    Sig.u <-reSigma  #random effect variance matrix
    BinvVB <- t(yZi) %*% yZi / sig.y^2
    Sig.u.inv <- solve(Sig.u)
    Hinv <- chol2inv(chol(BinvVB+Sig.u.inv))

# Get h vector
    yran <- yi - yXi%*%theta
    BinVyran <- sweep(yZi,1,yran,"*")
    BinVyran <- apply(BinVyran,2,sum) / sig.y^2
    h <- Hinv%*%BinVyran
    mu <-  h

# Linear combinations of random effects for L matrix
    renum<-dim(reSigma)[2]
    fixnum<-length(theta)
    L=Lpred=0
    Lpop=Lpoppred=0
    for(j in 1:length(gamma))
    {
        L=L+Lmat[,((j-1)*renum+1):(j*renum)]*gamma[j]
        Lpred=Lpred+Lpredmat[,((j-1)*renum+1):(j*renum)]*gamma[j]
        Lpop=Lpop+Lpopmat[,((j-1)*fixnum+1):(j*fixnum)]*gamma[j]
        Lpoppred=Lpoppred+Lpoppredmat[,((j-1)*fixnum+1):(j*fixnum)]*gamma[j]
    }
    if(class(L)=='numeric'){L=t(as.matrix(L))}
    if(class(Lpop)=='numeric'){Lpop=t(as.matrix(Lpop))}

# Get Skew-normal parameters
    nu <- - sXi%*%beta - L%*%h-Lpop%*%theta
    Sig11 <- Hinv
    Sig21 <- -L%*%Hinv
    Sig22 <- diag(nt) + L%*%Hinv%*%t(L)

# Generate Monte Carlo samples from skew-Normal distribution
    uall<-NULL
    for(l in 1:nMC){
```

```
        j <- 1
        omega <- rep(1,nt)
            breakflag <- F
            while(any(omega>0))
            {
            omega <- rmnorm(n=1, mean=nu, varcov=Sig22)
            j <- j+1
            if((j>jmax) & any(omega>0))
            {
                    breakflag <- T
                    break
            }
            }
            if(!breakflag)
            {
            mucond <- mu + t(Sig21) %*% solve(Sig22) %*% (omega - nu)
            Sigcond <- Sig11 - t(Sig21) %*% solve(Sig22) %*% Sig21
            u <- rmnorm(n=1, mean=mucond, varcov=Sigcond)
              uall<-rbind(uall,u)
            }
    }
    ustat1=apply(uall,2,mean)
    ustat2=apply(uall,2,median)
    ustat3=apply(uall,2, modeb)

# Linear predictors for surviving each time interval
    qq1<- newsXi%*%beta+Lpred%*%(ustat1)+Lpoppred%*%theta
    qq2<- newsXi%*%beta+Lpred%*%(ustat2)+Lpoppred%*%theta
    qq3<- newsXi%*%beta+Lpred%*%(ustat3)+Lpoppred%*%theta
# Predicted survival probabilities
    ppj1<-prod(pnorm(qq1[(nt+1):(nt+deltaTi)],lower.tail=T))
    ppj2<-prod(pnorm(qq2[(nt+1):(nt+deltaTi)],lower.tail=T))
    ppj3<-prod(pnorm(qq3[(nt+1):(nt+deltaTi)],lower.tail=T))

    out <- c(qnorm(ppj1), ppj1,qnorm(ppj2), ppj2,qnorm(ppj3), ppj3 )
    return(out)
}
```

The following code predicts the probability of surviving $\Delta T = 1$ intervals for all in the testing data set at each time interval they are at risk and calculates mean squared errors of survival predictions on the probit scale. Set `deltaT=2` or `deltaT=3` for the probability of surviving a further 2 or 3 time intervals respectively.

```
########  Set up data for prediction  ########

# Longitudinal data
knott<-knotti<-(0:11)/12
nknot<-nknoti<-length(knott)-1
test.sub=test.data$sub-750
test.y=test.data$yi
test.time<-test.data$ti
test.id<-unique(test.data$sub)
test.lambda<-test.data$lambdai

# Survival data
test.S=test.data$finalsurvi
trueS<-test.data$S
trueD<-test.data$D
test.sub2=test.sub
test.T<-test.data$visit  ## interval index
test.r<-(test.T-1)/11
test.nsurv<-length(test.T)
```

```
# Data for penalized spline model
test.X <-model.matrix(test.y~test.time)
test.Z <-outer(test.time,knott[-1],'-')
test.Z <-test.Z*(test.Z>0)

# Data for cubic spline model
test.X.bs <-model.matrix(test.y~test.time+I(test.time^2)+I(test.time^3))
test.Z.bs <-outer(test.time,c(0.25,0.5,0.75),'-')
test.Z.bs <-test.Z.bs^3*(test.Z.bs>0)


########  Extract parameters from fitted models  ########

# Survival model with time-varying covariate
surv.par<-as.matrix(coef(surv.fit.timev))
beta.timev<-as.matrix(surv.par[1:3,])
gamma.timev<-surv.par[4]

# Two-stage model
beta.hat<-fit.spline$coeff$fixed
u.hat<-unlist(fit.spline$coeff$random)
theta.2stage=c(beta.hat,u.hat[1:ncol(test.Z)])
beta.2stage<-coef(surv.spline)[1:3]
gamma.2stage<-coef(surv.spline)[4:5]
varmat<-VarCorr(fit.spline)
ySigma.2stage<-as.numeric(varmat[dim(varmat)[1],1])
sigma0.2stage=as.numeric(varmat[14,1])    # This is sigma0 squared
sigma1.2stage=as.numeric(varmat[15,1])  # This is sigma1 squared
sigma2.2stage=as.numeric(varmat[27,1])  # This is sigma2 squared
rho.2stage=as.numeric(varmat[15,3])
# RE variance
reSigma.2stage=Sigma.spline2(nknoti,c(sigma0.2stage,sigma1.2stage,sigma2.2stage,rho.2stage))

# Joint model with random intercept and slope
modelfit<-simfit.IS
est <- modelfit$estimate
ind.sigy <- which(names(est)=="sigmay")
ind.sig1 <- which(names(est)=="sigma1")
ind.rho <- which(names(est)=="rho")
ind.gam0 <- which(names(est)=="gamma0")
ind.gam1 <- which(names(est)=="gamma1")
# transform model parameters to original scales
estnew <- c(est[1:(ind.sigy-1)],exp(est[ind.sigy:ind.sig1]),frhoinv(est[ind.rho]),est[ind.gam0:ind.gam1])
theta.poplinear<-estnew[1:2]
beta.poplinear<-estnew[3:(ind.sigy-1)]
gamma.poplinear<-estnew[ind.gam0:ind.gam1]
ySigma.poplinear<-(estnew[ind.sigy])^2
sigma0.poplinear=(estnew[ind.sig1-1])^2 # This is sigma0 squared
sigma1.poplinear=(estnew[ind.sig1])^2   # This is sigma1 squared
rho.poplinear=estnew[ind.rho]
reSigma.poplinear=Sigma.is2(c(sigma0.poplinear,sigma1.poplinear,rho.poplinear))

# Joint model with penalized splines
modelfit<-simfit.PS
est <- modelfit$estimate
ind.sigy <- which(names(est)=="sigmay")
ind.sig1 <- which(names(est)=="sigma1")
ind.sigB <- which(names(est)=="sigmaB")
ind.rho <- which(names(est)=="rho")
ind.gam0 <- which(names(est)=="gamma0")
ind.gam1 <- which(names(est)=="gamma1")
# transform model parameters to original scales
estnew <- c(est[1:(ind.sigy-1)],exp(est[ind.sigy:ind.sig1]),exp(est[ind.sigB]),frhoinv(est[ind.rho]),
        est[ind.gam0:ind.gam1])
theta.spline=estnew[1:(2+11)]
beta.spline<-estnew[(3+11):(ind.sigy-1)]
gamma.spline<-estnew[ind.gam0:ind.gam1]
ySigma.spline<-(estnew[ind.sigy])^2
```

```
sigma0.spline=(estnew[ind.sig1-1])^2    # This is sigma0 squared
sigma1.spline=(estnew[ind.sig1])^2      # This is sigma1 squared
sigma2.spline=(estnew[ind.sigB])^2      # This is sigma2 squared
rho.spline=estnew[ind.rho]
reSigma.spline=Sigma.spline2(nknoti,c(sigma0.spline,sigma1.spline,sigma2.spline,rho.spline))

# Joint model with cubic splines
modelfit<-simfit.CS
est <- modelfit$estimate
ind.sigy <- which(names(est)=="sigmay")
ind.sig1 <- which(names(est)=="sigma1")
ind.sigB1 <- which(names(est)=="sigmaB1")
ind.sigB2 <- which(names(est)=="sigmaB2")
ind.sigB3 <- which(names(est)=="sigmaB3")
ind.gam0 <- which(names(est)=="gamma0")
ind.gam1 <- which(names(est)=="gamma1")
# transform model parameters to original scales
estnew <- c(est[1:(ind.sigy-1)],exp(est[ind.sigy:ind.sigB3]),est[ind.gam0:ind.gam1])
theta.Bspline=estnew[1:7]
beta.Bspline<-estnew[8:(ind.sigy-1)]
gamma.Bspline<-estnew[ind.gam0:ind.gam1]
ySigma.Bspline<-(estnew[ind.sigy])^2
sigmaB1.Bspline=(estnew[ind.sigB1 ])^2
sigmaB2.Bspline=(estnew[ind.sigB2 ])^2
sigmaB3.Bspline=(estnew[ind.sigB3 ])^2
reSigma.Bspline<-diag(c( (estnew[ind.sigB1:ind.sigB3])^2,  0.000237439^2, 0.0003074187^2,
            0.0009380569^2, 0.02841608^2))


########  Set up L matrices with linear combinations for survival model associations  ########

test.sX=cbind(1,test.r,test.r^2)

# L matrices for random intercept and slope model
Lint.linear<-matrix(0, test.nsurv, 2)  # random intercept at the start of the interval
Lint.linear[,1]<-1
Lint.linear[,2]<-knotti[test.T]
Lslope.linear<-matrix(0, test.nsurv, 2)  # random slope at the start of the interval
Lslope.linear[,2]<-1

# L matrices for penalized spline model
Lslope.spline<-Lint.spline<-matrix(0, test.nsurv, nknoti+2)
Lslope.spline[, 1]<-0
for (l in 2:(nknoti+2))# 1, ..., 11
{
  Lslope.spline[,l]<-as.numeric(test.T>=(l-1))
}
Lint.spline[,1]<-1
for (l in 2:(nknoti+1))# 1, ..., 11
{
  Lint.spline[,l]<-as.numeric(test.T>=l)*(knotti[test.T]-knotti[l-1])   # knotti now include zero
}

## L matrices for cubic spline model
Lslope.Bspline<-Lint.Bspline<-matrix(0, test.nsurv, 7)
bknots=c(0.25,0.5,0.75)
Lslope.Bspline[,1]<-0
Lslope.Bspline[,2]<-1
Lslope.Bspline[,3]<-2*knotti[test.T]
Lslope.Bspline[,4]<-3*(knotti[test.T])^2
Lslope.Bspline[,5]<-3*as.numeric(knotti[test.T]>bknots[1])*(knotti[test.T]-bknots[1])^2
Lslope.Bspline[,6]<-3*as.numeric(knotti[test.T]>bknots[2])*(knotti[test.T]-bknots[2])^2
Lslope.Bspline[,7]<-3*as.numeric(knotti[test.T]>bknots[3])*(knotti[test.T]-bknots[3])^2
Lint.Bspline[,1]<-1
Lint.Bspline[,2]<-knotti[test.T]
Lint.Bspline[,3]<-(knotti[test.T])^2
Lint.Bspline[,4]<-(knotti[test.T])^3
Lint.Bspline[,5]<-as.numeric(knotti[test.T]>bknots[1])*(knotti[test.T]-bknots[1])^3
```

```
Lint.Bspline[,6]<-as.numeric(knotti[test.T]>bknots[2])*(knotti[test.T]-bknots[2])^3
Lint.Bspline[,7]<-as.numeric(knotti[test.T]>bknots[3])*(knotti[test.T]-bknots[3])^3



########  Get survival predictions for all models  #########

predall=NULL
deltaT=1        # Number of intervals to predict survival for

for (i in 1:length(test.id) )
{
    surv.ni=max(test.T[test.sub2==i & test.T<=trueS & test.T<=trueD])       # last observed interval
    test.Si=test.S[test.sub2==i]
    event=test.Si[surv.ni]                  # status at last observed interval

    if (event==0 & surv.ni==1)
    {
# If event in first interval then no prediction to be done
        predall<-rbind(predall, c(test.id[i],rep(NA,20)))
    }else{

# Data for ith subject
        lambdai=test.lambda[test.sub==i]
        yi=test.y[test.sub==i]
        yXi=cbind(test.X[test.sub==i,],test.Z[test.sub==i,])
        yZi=yXi
        ytime=test.time[test.sub==i]
            yXi.bs<-cbind(test.X.bs[test.sub==i,],test.Z.bs[test.sub==i,])
        yZi.bs<-yXi.bs
        Ti=test.T[test.sub2==i]
        Si=test.S[test.sub2==i]
        sXi=test.sX[test.sub2==i,]
        Lpredmat.poplinear<-cbind(Lint.linear[test.sub2==i,],Lslope.linear[test.sub2==i,])
        Lpredmat.spline<-cbind(Lint.spline[test.sub2==i,],Lslope.spline[test.sub2==i,])
        Lpredmat.Bspline<-cbind(Lint.Bspline[test.sub2==i,],Lslope.Bspline[test.sub2==i,])
        Lpop.predmat.poplinear<-cbind(Lint.linear[test.sub2==i,],Lslope.linear[test.sub2==i,])
        Lpop.predmat.spline<-cbind(Lint.spline[test.sub2==i,],Lslope.spline[test.sub2==i,])
        Lpop.predmat.Bspline<-cbind(Lint.Bspline[test.sub2==i,],Lslope.Bspline[test.sub2==i,])

# Latest prediction interval
        if(event==1){maxinterval= min(surv.ni, 12-deltaT)}
        if(event==0 & surv.ni>1){maxinterval= min(surv.ni-1, 12-deltaT)}
        for(j in 1:maxinterval){            # Prediction interval
# Data up to current prediction interval
            etaij<-qnorm(prod(lambdai[(j+1):(j+deltaT)]))
            yiold<-yi[ytime<=knotti[j+1]]
                if(j==1){
                yXiold.poplinear<-t(as.matrix(yXi[ytime<=knotti[j+1],1:2]))
                yZiold.poplinear<-t(as.matrix(yZi[ytime<=knotti[j+1],1:2]))
                    yXiold.spline<-t(as.matrix(yXi[ytime<=knotti[j+1],]))
                yZiold.spline<-t(as.matrix(yZi[ytime<=knotti[j+1],]))
                    yXiold.Bspline<-t(as.matrix(yXi.bs[ytime<=knotti[j+1],]))
                yZiold.Bspline<-yXiold.Bspline
                sXiold<-t(as.matrix(sXi[1:j,]))
                sXinew<-as.matrix(sXi[1:(j+deltaT),])
                Tiold<-Ti[1:j]
                Lmat.poplinear.i<-t(as.matrix(Lpredmat.poplinear[1:j,]))
                Lpredmat.poplinear.i<-(as.matrix(Lpredmat.poplinear[1:(j+deltaT),]))
                Lmat.spline.i<-t(as.matrix(Lpredmat.spline[1:j,]))
                Lpredmat.spline.i<-(as.matrix(Lpredmat.spline[1:(j+deltaT),]))
                Lmat.Bspline.i<-t(as.matrix(Lpredmat.Bspline[1:j,]))
                Lpredmat.Bspline.i<-(as.matrix(Lpredmat.Bspline[1:(j+deltaT),]))
                Lpop.mat.poplinear.i<-t(as.matrix(Lpop.predmat.poplinear[1:j,]))
                Lpop.predmat.poplinear.i<-(as.matrix(Lpop.predmat.poplinear[1:(j+deltaT),]))
                Lpop.mat.spline.i<-t(as.matrix(Lpop.predmat.spline[1:j,]))
                Lpop.predmat.spline.i<-(as.matrix(Lpop.predmat.spline[1:(j+deltaT),]))
                Lpop.mat.Bspline.i<-t(as.matrix(Lpop.predmat.Bspline[1:j,]))
```

```
            Lpop.predmat.Bspline.i<-(as.matrix(Lpop.predmat.Bspline[1:(j+deltaT),]))
        }
        if(j>1 & length(yiold)==1)
        {
            yXiold.poplinear<-t(as.matrix(yXi[ytime<=knotti[j+1],1:2]))
            yZiold.poplinear<-t(as.matrix(yZi[ytime<=knotti[j+1],1:2]))
            yXiold.spline<-t(as.matrix(yXi[ytime<=knotti[j+1],]))
            yZiold.spline<-t(as.matrix(yZi[ytime<=knotti[j+1],]))
            yXiold.Bspline<-t(as.matrix(yXi.bs[ytime<=knotti[j+1],]))
            yZiold.Bspline<-yXiold.Bspline
            sXiold<-sXi[1:j,]
            sXinew<-sXi[1:(j+deltaT),]
            Tiold<-Ti[1:j]
            Lmat.poplinear.i<-Lpredmat.poplinear[1:j,]
            Lpredmat.poplinear.i<-Lpredmat.poplinear[1:(j+deltaT),]
            Lmat.spline.i<-Lpredmat.spline[1:j,]
            Lpredmat.spline.i<-Lpredmat.spline[1:(j+deltaT),]
            Lmat.Bspline.i<-Lpredmat.Bspline[1:j,]
            Lpredmat.Bspline.i<-Lpredmat.Bspline[1:(j+deltaT),]
            Lpop.mat.poplinear.i<-Lpop.predmat.poplinear[1:j,]
            Lpop.predmat.poplinear.i<-Lpop.predmat.poplinear[1:(j+deltaT),]
            Lpop.mat.spline.i<-Lpop.predmat.spline[1:j,]
            Lpop.predmat.spline.i<-Lpop.predmat.spline[1:(j+deltaT),]
            Lpop.mat.Bspline.i<-Lpop.predmat.Bspline[1:j,]
            Lpop.predmat.Bspline.i<-Lpop.predmat.Bspline[1:(j+deltaT),]
        }
        if(j>1  & length(yiold)>1)
        {
            yXiold.poplinear<-yXi[ytime<=knotti[j+1],1:2]
            yZiold.poplinear<-yZi[ytime<=knotti[j+1],1:2]
            yXiold.spline<-yXi[ytime<=knotti[j+1],]
            yZiold.spline<-yZi[ytime<=knotti[j+1],]
            yXiold.Bspline<-yXi.bs[ytime<=knotti[j+1],]
            yZiold.Bspline<-yXiold.Bspline
            sXiold<-sXi[1:j,]
            sXinew<-sXi[1:(j+deltaT),]
            Tiold<-Ti[1:j]
            Lmat.poplinear.i<-Lpredmat.poplinear[1:j,]
            Lpredmat.poplinear.i<-Lpredmat.poplinear[1:(j+deltaT),]
            Lmat.spline.i<-Lpredmat.spline[1:j,]
            Lpredmat.spline.i<-Lpredmat.spline[1:(j+deltaT),]
            Lmat.Bspline.i<-Lpredmat.Bspline[1:j,]
            Lpredmat.Bspline.i<-Lpredmat.Bspline[1:(j+deltaT),]
            Lpop.mat.poplinear.i<-Lpop.predmat.poplinear[1:j,]
            Lpop.predmat.poplinear.i<-Lpop.predmat.poplinear[1:(j+deltaT),]
            Lpop.mat.spline.i<-Lpop.predmat.spline[1:j,]
            Lpop.predmat.spline.i<-Lpop.predmat.spline[1:(j+deltaT),]
            Lpop.mat.Bspline.i<-Lpop.predmat.Bspline[1:j,]
            Lpop.predmat.Bspline.i<-Lpop.predmat.Bspline[1:(j+deltaT),]
        }

# Prediction for random intercept and slope joint model
        out.poplinear=predict.all.t.sim(yiold,yXiold.poplinear,yZiold.poplinear,sXinew,sXiold,Tiold,
            theta.poplinear,beta.poplinear,gamma.poplinear,Lmat.poplinear.i,Lpredmat.poplinear.i,
            Lpop.mat.poplinear.i,Lpop.predmat.poplinear.i,ySigma.poplinear,reSigma.poplinear,
            deltaT,200,100)
# Prediction for joint model with penalized splines
        out.spline=predict.all.t.sim(yiold,yXiold.spline,yZiold.spline,sXinew,sXiold,Tiold,
            theta.spline,beta.spline,gamma.spline,Lmat.spline.i,Lpredmat.spline.i,Lpop.mat.spline.i,
            Lpop.predmat.spline.i,ySigma.spline,reSigma.spline,deltaT,nMC=200,jmax=100)
# Prediction for joint model with cubic splines
        out.Bspline=predict.all.t.sim(yiold,yXiold.Bspline,yZiold.Bspline,sXinew,sXiold,Tiold,
            theta.Bspline,beta.Bspline,gamma.Bspline,Lmat.Bspline.i,Lpredmat.Bspline.i,
            Lpop.mat.Bspline.i,Lpop.predmat.Bspline.i,ySigma.Bspline,reSigma.Bspline,deltaT,
            nMC=200,jmax=100)
# Prediction for survival model with time-varying covariate
        out.timev=predict.all.t.sim.timev(yiold,sXinew,Tiold,beta.timev,gamma.timev,deltaT)
# Prediction for two-stage model
```

```
            out.spline2s=predict.all.t.sim.2stage(yiold,yXiold.spline,yZiold.spline,sXinew,sXiold,Tiold,
                theta.2stage,beta.2stage,gamma.2stage,Lmat.spline.i,Lpredmat.spline.i,Lpop.mat.spline.i,
                Lpop.predmat.spline.i,ySigma.2stage,reSigma.2stage,deltaT,200)

            predall<-rbind(predall, c(test.id[i],etaij,out.poplinear,out.spline,out.Bspline,out.timev,
                out.spline2s))
        }
    }
}

pred.all<-data.frame(unlist(predall))


########  Calculate mean-squared errors  ########

pred.all$error.poplinear1<-(pred.all$X3-pred.all$X2)^2
pred.all$error.poplinear2<-(pred.all$X5-pred.all$X2)^2
pred.all$error.poplinear3<-(pred.all$X7-pred.all$X2)^2

pred.all$error.spline1<-(pred.all$X9-pred.all$X2)^2
pred.all$error.spline2<-(pred.all$X11-pred.all$X2)^2
pred.all$error.spline3<-(pred.all$X13-pred.all$X2)^2

pred.all$error.Bspline1<-(pred.all$X15-pred.all$X2)^2
pred.all$error.Bspline2<-(pred.all$X17-pred.all$X2)^2
pred.all$error.Bspline3<-(pred.all$X19-pred.all$X2)^2

pred.all$error.timev<-(pred.all$X21-pred.all$X2)^2

pred.all$error.2stage.spline1<-(pred.all$X23-pred.all$X2)^2
pred.all$error.2stage.spline2<-(pred.all$X25-pred.all$X2)^2
pred.all$error.2stage.spline3<-(pred.all$X27-pred.all$X2)^2

pred.all$test.T<-test.T

# Overall MSE
meanerror<-rbind(meanerror, c(sim,deltaT,0,mean(pred.all$error.poplinear1, na.rm=T),
            mean(pred.all$error.spline1,na.rm=T),mean(pred.all$error.Bspline1,na.rm=T),
            mean(pred.all$error.poplinear2,na.rm=T),mean(pred.all$error.spline2,na.rm=T),
            mean(pred.all$error.Bspline2,na.rm=T),mean(pred.all$error.poplinear3,na.rm=T),
            mean(pred.all$error.spline3,na.rm=T),mean(pred.all$error.Bspline3,na.rm=T)))

# MSE by prediction time
for (j in (1+deltaT):12)
{
  meanerror<-rbind(meanerror, c(sim, deltaT,j, mean(pred.all$error.timev[pred.all$test.T==j], na.rm=T),
            mean(pred.all$error.2stage.spline1[pred.all$test.T==j], na.rm=T),
            mean(pred.all$error.poplinear1[pred.all$test.T==j], na.rm=T),
            mean(pred.all$error.spline1[pred.all$test.T==j], na.rm=T),
            mean(pred.all$error.Bspline1[pred.all$test.T==j], na.rm=T),
            mean(pred.all$error.poplinear2[pred.all$test.T==j], na.rm=T),
            mean(pred.all$error.spline2[pred.all$test.T==j], na.rm=T),
            mean(pred.all$error.Bspline2[pred.all$test.T==j], na.rm=T),
            mean(pred.all$error.poplinear3[pred.all$test.T==j], na.rm=T),
            mean(pred.all$error.spline3[pred.all$test.T==j], na.rm=T),
            mean(pred.all$error.Bspline3[pred.all$test.T==j], na.rm=T)))
}
```
