# Supplementary material for: Dynamic predictions using flexible joint models of longitudinal and time‐to‐event data
Source: Stat Med. 2017 Jan 22;36(9):1447–60. doi: 10.1002/sim.7209 (PMC5381717; doi:10.1002/sim.7209)

Supplementary Materials to 'Dynamic Predictions using Flexible Joint Models of Longitudinal and Time-to-event Data'

Jessica Barrett
Department of Public Health and Primary Care, University of Cambridge, Strangeways Research Laboratory, Worts Causeway, Cambridge CB1 8RN, UK
email: jkb23@medschl.cam.ac.uk

and

Li Su[1]
MRC Biostatistics Unit, Robinson Way, Cambridge CB2 0SR, U.K.
email: li.su@mrc-bsu.cam.ac.uk

1 Evaluation of the marginal likelihood

Barrett *et al.* showed that the likelihood in (7) of the paper can be written in a closed form using the multivariate skew-Normal results for integrating out $\mathbf{b}_i$ [1, 2]. Let $\mathbf{H}_i = \mathbf{B}_i^{\mathrm{T}}\mathbf{V}_i^{-1}\mathbf{B}_i + \boldsymbol{\Sigma}^{-1}$, $\mathbf{h}_i = \mathbf{H}_i^{-1}\mathbf{B}_i^{\mathrm{T}}\mathbf{V}_i^{-1}(\mathbf{Y}_i - \mathbf{x}_i\boldsymbol{\alpha} - \mathbf{B}_i\boldsymbol{\beta})$ and

$$\begin{aligned}\mathcal{L}_{1i}(\boldsymbol{\theta} \mid \mathbf{Y}_i) &= \exp\{-\log(2\pi)n_i/2 - \log(|\mathbf{V}_i|)/2 - \log(|\boldsymbol{\Sigma}\mathbf{H}_i|)/2 \\ &\quad -(\mathbf{Y}_i - \mathbf{x}_i\boldsymbol{\alpha} - \mathbf{B}_i\boldsymbol{\beta})^{\mathrm{T}}\mathbf{V}_i^{-1}(\mathbf{Y}_i - \mathbf{x}_i\boldsymbol{\alpha} - \mathbf{B}_i\boldsymbol{\beta})/2 + \mathbf{h}_i^{\mathrm{T}}\mathbf{H}_i\mathbf{h}_i/2\}.\end{aligned} \quad (1)$$

Then we have

$$f(\mathbf{Y}_i \mid \mathbf{b}_i; \boldsymbol{\theta})f(\mathbf{b}_i; \boldsymbol{\theta}) = \phi^{(M+1)}(\mathbf{b}_i; \mathbf{h}_i, \mathbf{H}_i^{-1})\mathcal{L}_{1i}(\boldsymbol{\theta} \mid \mathbf{Y}_i),$$

where $\phi^{(M+1)}(\mathbf{b}_i; \mathbf{h}_i, \mathbf{H}_i^{-1})$ is the $(M+1)$-dimensional multivariate Normal density with mean $\mathbf{h}_i$ and variance matrix $\mathbf{H}_i^{-1}$. Further, we define $\tilde{\mathbf{X}}_{ir} = (\tilde{\mathbf{x}}_{i1}, \ldots, \tilde{\mathbf{x}}_{ir})^{\mathrm{T}}$ and $\mathbf{L}_{ir} = (\mathbf{W}_{i1}^{\mathrm{T}}\boldsymbol{\gamma}_1, \ldots, \mathbf{W}_{ir}^{\mathrm{T}}\boldsymbol{\gamma}_r)^{\mathrm{T}}$ for $r = 1, \ldots, s$. Then (8) in the paper can be written as

$$f(s, \delta_i \mid \mathbf{b}_i; \boldsymbol{\theta}) = \begin{cases} \Phi^{(s)}(\tilde{\mathbf{X}}_{is}\tilde{\boldsymbol{\alpha}} + \mathbf{L}_{is}\mathbf{b}_i) & \delta_i = 0, \\ \Phi^{(s-1)}(\tilde{\mathbf{X}}_{i,s-1}\tilde{\boldsymbol{\alpha}} + \mathbf{L}_{i,s-1}\mathbf{b}_i) - \Phi^{(s)}(\tilde{\mathbf{X}}_{is}\tilde{\boldsymbol{\alpha}} + \mathbf{L}_{is}\mathbf{b}_i) & \delta_i = 1, \end{cases}$$

where $\Phi^{(r)}(\cdot) = \Phi^{(r)}(\cdot; \mathbf{0}, \mathbf{I})$ is the standard r-dimensional multivariate Normal cumulative distribution function. Next we write $\mathcal{L}_i(\boldsymbol{\theta} \mid \mathbf{Y}_i, s, \delta_i) = \mathcal{L}_{1i}(\boldsymbol{\theta} \mid \mathbf{Y}_i)\mathcal{L}_{2i}(\boldsymbol{\theta} \mid \mathbf{Y}_i, s, \delta_i)$, where

[1] Joint first author.

$\mathcal{L}_{2i}(\boldsymbol{\theta} \mid \mathbf{Y}_i, s, \delta_i) = \int f(s, \delta_i \mid \mathbf{b}_i; \boldsymbol{\theta})\Phi^{(M+1)}(\mathbf{b}_i; \mathbf{h}_i, \mathbf{H}_i^{-1})d\mathbf{b}_i$. Using the multivariate skew-Normal results [1, 2], it can be shown that

$$\mathcal{L}_{2i}(\boldsymbol{\theta} \mid \mathbf{Y}_i, s, \delta_i) = \begin{cases} \Phi^{(s)}\left(\tilde{\mathbf{X}}_{is}\tilde{\boldsymbol{\alpha}} + \mathbf{L}_{is}\mathbf{h}_i; \mathbf{0}, \mathbf{I} + \mathbf{L}_{is}\mathbf{H}_i^{-1}\mathbf{L}_{is}^{\mathrm{T}}\right) & \delta_i = 0, \\ \Phi^{(s-1)}\left(\tilde{\mathbf{X}}_{is-1}\tilde{\boldsymbol{\alpha}} + \mathbf{L}_{i,s-1}\mathbf{h}_i; \mathbf{0}, \mathbf{I} + \mathbf{L}_{i,s-1}\mathbf{H}_i^{-1}\mathbf{L}_{i,s-1}^{\mathrm{T}}\right) & \\ -\Phi^{(s)}\left(\tilde{\mathbf{X}}_{is}\tilde{\boldsymbol{\alpha}} + \mathbf{L}_{is}\mathbf{h}_i; \mathbf{0}, \mathbf{I} + \mathbf{L}_{is}\mathbf{H}_i^{-1}\mathbf{L}_{is}^{\mathrm{T}}\right) & \delta_i = 1. \end{cases}$$

We use the `R` package `mnormt` for numerical evaluation of the multivariate Normal probabilities in $\mathcal{L}_{2i}(\boldsymbol{\theta} \mid \mathbf{Y}_i, s, \delta_i)$.

2 Posterior distribution of $\mathbf{b}_i$

It is easy to see that the posterior distribution of the individual-level P-spline coefficients $\mathbf{b}_i$ used in the dynamic prediction procedure is

$$f(\mathbf{b}_i \mid S_i > r, \mathbf{Y}_i\{t(r)\}, \hat{\boldsymbol{\theta}}, \lambda) \propto \phi^{(M+1)}(\mathbf{b}_i; \hat{\mathbf{h}}_i, \hat{\mathbf{H}}_i)\Phi^{(r)}(\tilde{\mathbf{X}}_{ir}\hat{\tilde{\boldsymbol{\alpha}}} + \hat{\mathbf{L}}_{ir}\mathbf{b}_i),$$

which follows the form of a multivariate skew-Normal distribution [1]. That is,

$$\begin{aligned} & f(\mathbf{b}_i \mid S_i > r, \mathbf{Y}_i\{t(r)\}, \hat{\boldsymbol{\theta}}, \lambda) \\ = \; & |\boldsymbol{\Sigma}_{11}|^{-1/2}\phi^{(M+1)}(\boldsymbol{\Sigma}_{11}^{-1/2}(\mathbf{b}_i - \bar{\boldsymbol{\mu}}_1); \mathbf{0}, \mathbf{I})\frac{\Phi^{(r)}(-\bar{\boldsymbol{\mu}}_2 - \boldsymbol{\Sigma}_{21}\boldsymbol{\Sigma}_{11}^{-1}(\mathbf{b}_i - \bar{\boldsymbol{\mu}}_1); \mathbf{0}, \mathbf{I})}{\Phi^{(r)}(-\bar{\boldsymbol{\mu}}_2; \mathbf{0}, \boldsymbol{\Sigma}_{22})} \end{aligned} \quad (2)$$

where $\boldsymbol{\Sigma}_{11} = \hat{\mathbf{H}}_i$, $\boldsymbol{\Sigma}_{21} = -\hat{\mathbf{L}}_{ir}\hat{\mathbf{H}}_i^{-1}$, $\boldsymbol{\Sigma}_{22} = \mathbf{I} + \hat{\mathbf{L}}_{ir}\hat{\mathbf{H}}_i^{-1}(\hat{\mathbf{L}}_{ir})^{\mathrm{T}}$, $\bar{\boldsymbol{\mu}}_1 = \hat{\mathbf{h}}_i$, $\bar{\boldsymbol{\mu}}_2 = -\tilde{\mathbf{X}}_{ir}\hat{\tilde{\boldsymbol{\alpha}}} - \hat{\mathbf{L}}_{ir}\hat{\mathbf{h}}_i$. Sampling from (2) can proceed by drawing from $\bar{\mathbf{y}} \sim N^{(r)}(\bar{\boldsymbol{\mu}}_2, \boldsymbol{\Sigma}_{22})$ until $\bar{\mathbf{y}} \leq \mathbf{0}$, then draw $\mathbf{b}_i^{(l)}$ from $N^{(M+1)}(\bar{\boldsymbol{\mu}}_1 + \boldsymbol{\Sigma}_{21}^{\mathrm{T}}\boldsymbol{\Sigma}_{22}^{-1}(\bar{\mathbf{y}} - \bar{\boldsymbol{\mu}}_2), \boldsymbol{\Sigma}_{11} - \boldsymbol{\Sigma}_{21}^{\mathrm{T}}\boldsymbol{\Sigma}_{22}^{-1}\boldsymbol{\Sigma}_{21})$.

3 Additional results for the HERS data example

Figure 2 presented the dynamic predicted survival probabilities for HERS patient 26 at the probit scale.

4 Simulation study

In this section, we perform a simulation study to evaluate the finite sample performance of the proposed flexible joint model. Specifically, we will compare its performance in dynamic prediction with (1) a survival model using observed longitudinal outcome as a time-varying covariate; (2) a two-stage approach that uses empirical Bayes estimates of random effects, based on a linear mixed model with P-splines fitted to observed longitudinal data, in a subsequent survival model; (3) a joint model with random intercept and slope only; and (4) a joint model with cubic splines and 3 internal knots [3]. Except for the survival model with observed longitudinal outcome as a time-varying covariate, the same survival sub-model is specified for the three joint models as well as in the two-stage approach. We will use the 'gold standard' estimator of $\pi_i(s \mid r)$ with the true (i.e. simulated) values for random effects and true values for the parameters and evaluate the dynamic prediction accuracy as a function of the follow-up time interval r (corresponding to the different amounts of longitudinal data available for prediction for an individual) and also the prediction window $\Delta t = s - r$.

4.1 Design

The design of the simulation study is motivated by the HERS data in Section 4 of the main text. Specifically, we assume that the mean function of the longitudinal process is $\mu_i(t) = m_i(t)$ and does not depend on other covariates. In order to decide the true $m_i(t)$, we first fit a non-linear model

$$\mu(t) = \frac{\sin\{2\pi a(t+b)\}}{\{1+3(t+b)^3\}} + c + d \cdot t$$

to the HERS data using non-linear least squares estimator (*nls* function in R) assuming the data are independent, and obtain the estimates $\hat{a} = 0.6343, \hat{b} = 0.7685, \hat{c} = 0.2283, \hat{d} = -0.1924$. Figure 3 presents the fitted curves to the HERS data by non-linear least squares and smoothing splines. Next we specify individual trajectories $m_i(t)$ as follows:

$$m_i(t) = b_{i1}\frac{\sin\{2\pi\hat{a}(t+\hat{b})\}}{\{1+3(t+\hat{b})^3\}} + (\hat{c} + b_{i0}) + \{\hat{d} + (b_{i1} - 1)/0.3\}t,$$

where $b_{i0} \sim N(0, 1)$ and $b_{i1} \sim N(1, 0.3^2)$ with correlation $\rho = -0.2$. As a result, $\mathrm{E}_{b_{i0}, b_{i1}}\{m_i(t)\} = \mu(t)$. Figure 4 presents several true $m_i(t)$ given b_{i0}, b_{i1}.

We divide $[0, 1]$ into 12 equally spaced intervals separated by boundary points $k_0 = 0, k_1, k_2, \ldots k_{11}, k_{12} = 1$ and simulate the measurement time t_{ir} uniformly within the rth interval ($r = 1, \ldots, 12$). For each patient, first we simulate b_{i0}, b_{i1} and obtain $m_i(t)$ as the mean function. Then we simulate $Y_{ir} \sim N(m_i(t_{ir}), 0.16)$. The complete longitudinal data for each patient contain 12 observations.

The true survival sub-model is

$$\mathrm{P}(S_i = r \mid S_i \geq r) = 1 - \Phi\left\{\alpha_0 + \alpha_1 r^* + \alpha_2 r^{*2} + \gamma_0 m_i(k_{r-1}) + \gamma_1 m_i'(k_{r-1})\right\} \qquad (3)$$

for $r = 1, \ldots, 12$, where $r^* = (r-1)/11$ and $\alpha_0 = 3$, $\alpha_1 = -2.5$, $\alpha_2 = 2$, $\gamma_0 = 0.7$, $\gamma_1 = 0.2$. That is, the discrete hazard at the rth interval is associated with the value and the 1st derivative of $m_i(t)$ at the starting point of the rth interval k_{r-1}.

For censoring other than deaths, we use the Kaplan-Meier estimates of the censoring (dropout) probability from the HERS data to simulate the censoring distribution. Each simulated dataset contains data from 850 patients and on average we obtain 7550 longitudinal observations per simulation, which is similar to the censoring percentage 69% in the HERS data.

In each simulation, we use data from 750 patients as the training set and fit the 5 models in comparison. The data from the remaining 100 patients are left as the testing set to evaluate dynamic prediction accuracy. Under the above settings, we simulate 200 datasets.

The three joint models all have the same structure for survival sub-model as in (3), but the formulas for $m_i(k_{r-1})$ and $m_i'(k_{r-1})$ are different because of the different longitudinal sub-models. Specifically, we assume the following longitudinal sub-models:

1. Joint Model with random intercept and slope only (JM1):

$$m_i(t) = \beta_0 + b_{0i} + (\beta_1 + b_{1i}) \cdot t,$$

$$\begin{bmatrix} b_{i0} \\ b_{i1} \end{bmatrix} \sim N\left(\mathbf{0}, \begin{bmatrix} \sigma_0^2 & \rho\sigma_0\sigma_1 \\ \rho\sigma_0\sigma_1 & \sigma_1^2 \end{bmatrix}\right).$$

2. Joint Model with P-splines (JM2):

$$m_i(t) = \sum_{l=0}^{12} (\beta_l + b_{il}) B_l(t),$$

where $B_l(t)$ are the truncated linear basis with internal knots at $k_1, \ldots, k_{11}$ and

$$\begin{bmatrix} b_{i0} \\ b_{i1} \\ b_{i2} \\ \vdots \\ b_{i12} \end{bmatrix} \sim N\left(\mathbf{0}, \mathbf{\Sigma} = \begin{bmatrix} \sigma_0^2 & \rho\sigma_0\sigma_1 & \mathbf{0} \\ \rho\sigma_0\sigma_1 & \sigma_1^2 & \mathbf{0} \\ \mathbf{0} & \mathbf{0} & \sigma_2^2\mathbf{I}_{11} \end{bmatrix}\right).$$

For computational efficiency, we fix λ at 3.232, which is obtained by averaging the estimate of λ from fitting a linear mixed model with P-splines to the complete longitudinal data.

3. Joint Model with cubic splines (JM3):

$$m_i(t) = \sum_{l=0}^{6} (\beta_l + b_{il}) BS_l(t),$$

where $\{BS_l(t)\} = \{1, t, t^2, t^3, (t-0.25)_+^3, (t-0.5)_+^3, (t-0.75)_+^3\}$ is the truncated cubic basis with 3 internal knots at $0.25, 0.5, 0.75$ and $(a)_+^3 = a^3 \cdot \mathrm{I}(a \geq 0)$. Knot locations here were not based on percentiles of sample observation times, as is common practice, to avoid any dependence of the longitudinal sub-model on the survival process. Following [3], we assume that $b_{il} \sim N(0, \sigma_l^2)$ are independent $(l = 0, \ldots, 6)$. Preliminary results from a cubic spline model fitted to the complete longitudinal data revealed that the variances of some cubic random coefficients are very small. For computational efficiency, we therefore fix these variances at their average estimates from the complete longitudinal data.

In each simulation, we use the dynamic prediction procedure described in Section 3.3 of the main text to estimate $\pi_i(s \mid r)$ for the 100 patients in the testing set. Depending on the value of $\Delta t = s - r$, we will make predictions at the first interval $(r = 1)$ up to the 11th interval for $\Delta t = 1$, up to the 10th interval for $\Delta t = 2$, and up to the 9th interval for

$\Delta t = 3$. We will obtain the mean, median and mode estimates of the posterior random effect distributions, and plug them in to calculate $\hat{\pi}_i(s \mid r, \hat{\boldsymbol{\theta}})$.

The survival model with the time-varying covariate will follow the same structure as in (3), but Y_{ir}, instead of estimates of $m_i(k_{r-1})$ and $m'_i(k_{r-1})$, is incorporated as a time-varying covariate. The dynamic prediction procedure for this approach is similar to those used for the joint models, except that the last observed outcome Y_{ir} (instead of random effect estimates) is used in the fitted survival model for prediction.

In the two-stage approach, first we fit a linear mixed model with P-splines to the observed longitudinal data using the same specification as for JM2. The computation is done by the `lme` function in the R package `nlme`. Then using the empirical Bayes estimates of the random effects from the fitted linear mixed model, we fit a survival model with the same specification as in (3). Based on the parameter point estimates from the linear mixed model, we obtain the empirical Bayes estimates of the random effects for the patients in the testing set. Finally, using these random effect estimates and the fitted survival model, we produce predicted survival probabilities over time for the patients in the testing set. Note that, unlike in the joint models, the posterior distribution of the random effects used to generate empirical Bayes estimates in the two-stage approach will not involve the observed survival data.

4.2 Results

4.2.1 Bias and mean squared errors of parameter estimates

Table 1 gives the bias, empirical standard deviation (SD), mean squared error (MSE) and coverage probability for parameter estimates in the survival models from the two-stage approach and joint models. Overall, the joint model with P-splines (JM2) had good coverage probabilities and smaller biases. All models seem to have difficulty in estimating α_1 and α_2, possibly due to the the fact that the true survival rate is set quite high (e.g., $\alpha_0 = 3$), therefore there is not much information from the observed data to estimate the time trend in the survival rate accurately. For γ_0 and γ_1, JM2 has the smallest bias. The two-stage approach,

JM1 and JM3 had small biases for γ_0, but had large biases for γ_1. Specifically, the two-stage approach underestimate γ_1, which is not surprising because the two-stage approach tends to overestimate $m_i'(k_{r-1})$ by ignoring the selection of survival and using observed longitudinal data from patients with higher change rates in $m_i(t)$. On the other hand, JM1 and JM3 overestimate γ_1, possibly because the non-linearity in $m_i(t)$ are not captured very well.

Figure 5 shows the mean estimates and mean squared errors of the population curve $\mu(t)$ (with grid points of 0.01 in $[0, 1]$) from the three joint models and the two-stage approach. The estimated curves from the two-stage approach, JM2 and JM3 are similar and unbiased up to around $t = 0.2$. Beyond $t = 0.2$, the estimated curve from JM2 is close to the true curve. On the other hand, not surprisingly, the two-stage approach gradually overestimates $\mu(t)$ because it uses the observed longitudinal data only and ignores the selection by survival when t becomes larger. This is consistent with the underestimate of γ_1 in the two-stage approach. The estimated curve from JM3 underestimates $\mu(t)$ at large t, corresponding to the overestimate of γ_1. JM1 does not capture the non-linearity of $\mu(t)$, and it also underestimates $\mu(t)$ at large t, possibly due to the overestimate of γ_1. JM2 with P-splines also has the smallest mean squared error over t.

4.2.2 Dynamic predictions

We compare the predictions from the 5 models with the 'gold standard' of $\pi_i(s \mid r)$ based on the true values of the parameters and random effects. At the probit scale of the predicted probability, we calculate the root mean squared prediction errors (RMSE) of the predictions from the 5 models for each simulation as a function of the prediction time r and prediction window Δt.

Figures 6-8 present the box-plots of RMSE from the survival model with the time-varying covariate, the two-stage approach, and the three joint models using different sample summaries (mean, median, mode) of the posterior distribution of the random effects at different values of r and Δt. Table 2 gives the frequencies of the ranks of the RMSE aggregated over all prediction times (rank 1: lowest RMSE; rank 2: second lowest RMSE; rank 3: third

lowest RMSE; rank 4: fourth lowest RMSE; rank 5: highest RMSE) from the survival model with the time-varying covariate, the two-stage approach, and the three joint models with different sample summaries of random effect posterior distribution. Due to the sizes of the tables, results for individual prediction times r are not shown.

We summarize our findings as follows:

(1) JM2 (with P-splines) overall outperforms the other two joint models, regardless of the sample summaries used for random effect distribution. When the RMSE are calculated by aggregating all prediction times for each value of Δt, the medians of the RMSE across all simulations are lower in JM2 (see the top-left panels of Figures 6-8).

(2) The survival model with the time-varying covariate performs the worst among all models. Its RMSEs are constantly higher than those from other models for almost all prediction times except $r = 1$. This is not surprising since this survival model is misspecified.

(3) The two-stage approach also outperforms JM1 and JM3, only slightly worse than JM2. The frequency of having the lowest RMSE (aggregated over all prediction times) for the two-stage approach is only slightly lower than that from JM2. This phenomenon is very interesting because although the two-stage approach does not estimate the parameters and curves well, this has much less impact on its dynamic prediction performance.

(4) Depending on the shape of the true longitudinal trajectory $m_i(t)$ and prediction time r, JM1 (with random intercept and slope) and JM3 (with cubic splines) can perform similarly to JM2 (with P-splines). For example, when $\Delta t = 1$ and $r = 7$ or $r = 8$, the RMSEs of all joint models are very close. This might be because the estimated longitudinal trajectories around $r = 7$ or $r = 8$ from the joint models happen to be similar by design.

(5) JM3 (with cubic splines) performs the worst among the three joint models, especially at later prediction times r, and with much less accuracy (larger median of RMSE). In

addition, the variabilities of RMSE from JM3 are higher than JM1 and JM2 when $r \geq 8$. This might be explained by the wild behavior of the cubic splines when extrapolated to the next time interval for prediction [4, Chapter 5]. When less information is available for estimating the conditional survival probabilities at later prediction times $r \geq 7$, this problem is exacerbated.

(6) The survival mode with time-varying covariate and the two-stage approach have smaller variabilities in their prediction errors at later predictor times than the joint models. This is not surprising since joint models are more complex such that more variability is introduced into the estimation.

(7) Consistent with the findings in [5], for all joint models the variability of the prediction errors (interquartile range of RMSE) initially increases as r increases, but tends to decrease as the prediction time r progresses.

(8) Interestingly, the predictions based on the sample mode of the posterior distribution of random effects (similar to empirical Bayes estimates) are slightly worse than corresponding predictions based on the sample mean and median across all models.

In summary, both the two-stage approach and the joint model based on P-splines perform well in dynamic prediction, although their performances in parameter estimation are very different. Complex joint models with cubic splines might introduce strong prediction errors because of the strong extrapolation imposed.

References

1. Arnold BC. Flexible univariate and multivariate models based on hidden truncation. *Journal of Statistical Planning and Inference* 2009; **139**:3741–3749.

2. Barrett J, Diggle P, Henderson R, Taylor-Robinson D. Joint modelling of repeated measurements and time-to-event outcomes: flexible model specification and exact likelihood

inference. *Journal of the Royal Statistical Society: Series B (Statistical Methodology)* 2015; **77**:131–148, doi:10.1111/rssb.12060.

3. Rizopoulos D. *Joint Models for Longitudinal and Time-to-Event Data, with Applications in R.* Boca Raton: Chapman and Hall/CRC, 2012.

4. Hastie T, Tibshirani R, Friedman J. *The Elements of Statistical Learning: Data Mining, Inference, and Prediction.* Springer, 2009.

5. Rizopoulos D. Dynamic predictions and prospective accuracy in joint models for longitudinal and time-to-event data. *Biometrics* 2011; **67**:819–829, doi:10.1111/j.1541-0420.2010.01546.x.

Table 1: Bias, empirical standard deviation (SD), mean squared error (MSE) and coverage probability for parameter estimates in the survival models from the two-stage approach and joint models fitted to the 200 simulated datasets.

	True value	Empirical mean	Percent bias	Absolute bias	Empirical SD	MSE	Coverage probability
**2-stage**							
α_0	3	2.718	-9	-0.282	0.132	0.097	41
α_1	-2.5	-1.781	-29	0.719	0.517	0.783	76.5
α_2	2	1.424	-29	-0.576	0.509	0.590	81.5
γ_0	0.7	0.668	-5	-0.032	0.051	0.004	92.5
γ_1	0.2	0.077	-62	-0.123	0.038	0.017	22.5
**JM1**							
α_0	3	2.844	-5	-0.156	0.146	0.046	79
α_1	-2.5	-1.825	-27	0.675	0.575	0.785	78.5
α_2	2	1.715	-14	-0.285	0.636	0.484	93
γ_0	0.7	0.723	3	0.023	0.060	0.004	96
γ_1	0.2	0.299	50	0.099	0.071	0.015	76.5
**JM2**							
α_0	3	2.980	-1	-0.020	0.173	0.030	94.5
α_1	-2.5	-2.649	6	-0.149	0.651	0.444	98
α_2	2	2.368	18	0.368	0.703	0.627	94
γ_0	0.7	0.712	2	0.012	0.057	0.003	96
γ_1	0.2	0.208	4	0.008	0.070	0.005	96
**JM3**							
α_0	3	3.229	8	0.229	0.227	0.104	86.5
α_1	-2.5	-3.477	39	-0.977	0.827	1.636	83
α_2	2	3.083	54	1.083	0.848	1.889	79
γ_0	0.7	0.704	1	0.004	0.058	0.003	97
γ_1	0.2	0.266	33	0.066	0.069	0.009	87

Table 2: Frequency of the ranks of the root mean squared errors (RMSE) of the predicted probabilities (at probit scale) of surviving the next 1-3 intervals ($\Delta t = 1, 2, 3$) from the survival model with time-varying covariate (TY), the two-stage approach (2S), the three fitted joint models (JM1: with random intercept and slope only; JM2: with P-splines; JM3: with cubic splines) versus the gold standard based on the true values for the parameters and the random effects. Different estimates (mean, median, mode) based on 200 samples from the posterior distribution of random effects $\mathbf{b}_i$ are used for JM1-JM3. Results are based on 200 simulated datasets and RMSE are calculated for all prediction intervals ($\forall r$).

Δt	Rank	TY	2S	JM1	JM2	JM3	TY	2S	JM1	JM2	JM3	TY	2S	JM1	JM2	JM3
				Mean of $\mathbf{b}_i$					Median of $\mathbf{b}_i$					Mode of $\mathbf{b}_i$		
1	1	0	**69**	28	**76**	27	0	**69**	26	**77**	28	0	**89**	21	**61**	29
	2	23	16	51	72	38	23	18	50	72	37	31	18	43	79	29
	3	16	14	67	45	58	17	15	70	45	53	23	21	63	53	40
	4	22	101	43	7	27	23	98	44	6	29	18	72	62	7	41
	5	**139**	0	11	0	**50**	**137**	0	10	0	**53**	**128**	0	11	0	**61**
2	1	0	**61**	20	**83**	36	0	**64**	24	**80**	32	0	**89**	20	**66**	25
	2	21	20	49	71	39	21	18	45	72	44	29	19	38	81	33
	3	11	19	74	40	56	14	19	71	42	54	21	15	61	47	56
	4	16	100	47	6	31	15	99	49	6	31	16	77	62	6	39
	5	**152**	0	10	0	**38**	**150**	0	11	0	**39**	**134**	0	19	0	**47**
3	1	0	**55**	25	**81**	39	0	**56**	24	**81**	39	0	**89**	18	**61**	32
	2	20	22	40	79	39	20	25	40	76	39	29	24	30	83	34
	3	14	21	73	36	56	14	21	73	37	55	17	14	62	49	58
	4	10	102	51	4	33	11	98	53	6	32	18	73	70	6	33
	5	**156**	0	11	0	**33**	**155**	0	10	0	**35**	**136**	0	20	1	**43**

Figure 1: Diagrams explaining equations (3) and (4) from the main paper.

(a) Equation (3): Example plot of the contribution $\sum_{l=0}^{M} b_{il} B_l(t)$ from the random effects $\mathbf{b}_i$ to the mean function $\mu_i(t)$. The random intercept of the first interval is $W_{10}(\mathbf{b}_i) = b_{i0}$, of the second interval is $W_{20}(\mathbf{b}_i) = b_{i0} + \Delta_1 = b_{i0} + b_{i1}k$, of the third interval is $W_{30}(\mathbf{b}_i) = b_{i0} + \Delta_1 + \Delta_2 = b_{i0} + b_{i1}k_2 + b_{i2}(k_2 - k_1)$, and of the fourth interval is $W_{40}(\mathbf{b}_i) = b_{i0} + \Delta_1 + \Delta_2 + \Delta_3 = b_{i0} + b_{i1}k_3 + b_{i2}(k_3 - k_1) + b_{i3}(k_3 - k_2)$. Generalising to the rth interval gives equation (3).

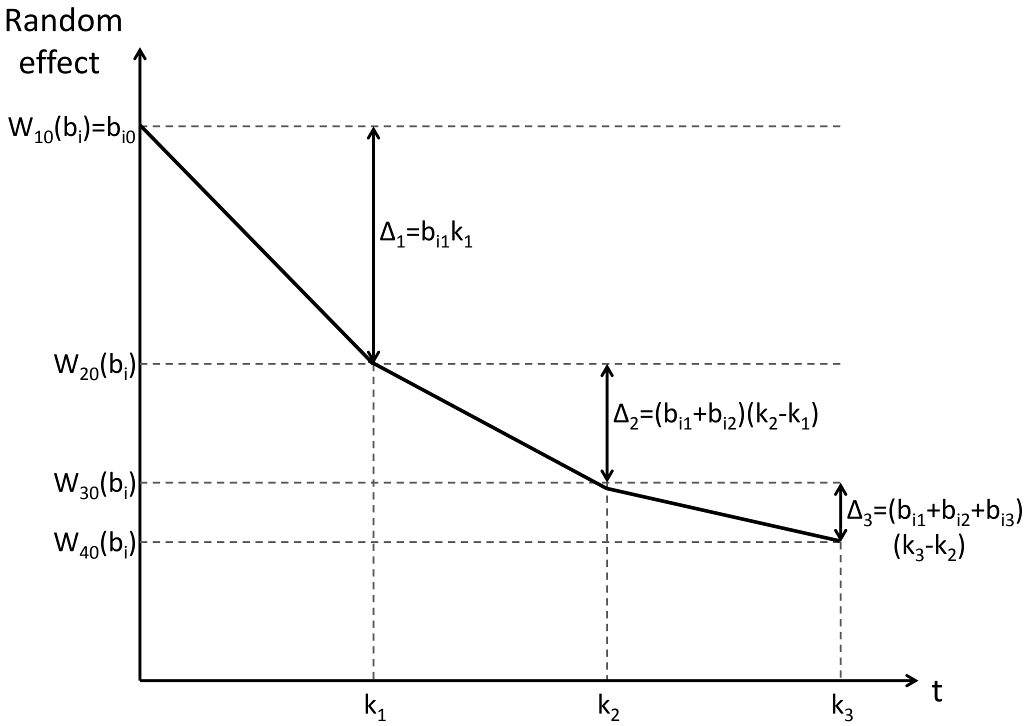

(b) Equation (4): Example plot demonstrating equation (4). The random effect b_{ir}, $r \geq 2$, is the change in slope between the $(r-1)$th interval and the rth interval.

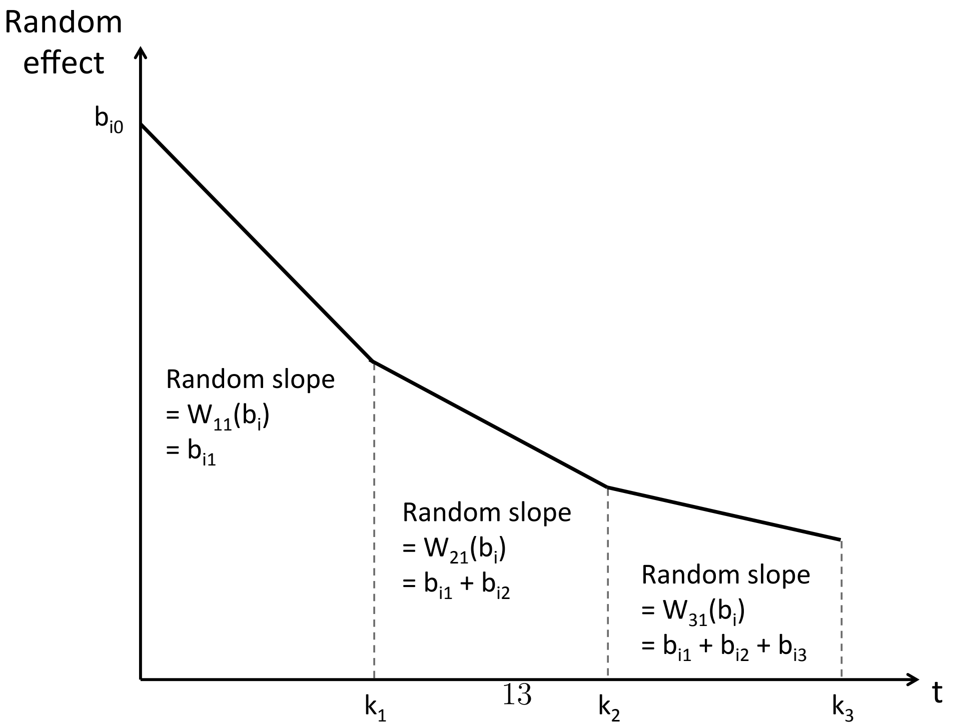

Figure 2: Left side of each panel: observed (standardized) square root CD4 counts and estimated individual longitudinal trajectory for Patient 26 up to the prediction time $r = 3, 5, 7, 9$. The dotted lines are the cut-off time for the prediction. The solid and dashed dark lines are estimated individual longitudinal trajectories (using medians of 200 samples from the posterior of $\mathbf{b}_i$) based on Model 1 and Model 2, respectively. Right side of each panel: predicted conditional probabilities of HIV survival (at probit scale) after the next 1, 2 and 3 time intervals. Squares represent predictions from Model 1 and triangles represent predictions from Model 2 (using medians of 200 samples from the posterior of $\mathbf{b}_i$).

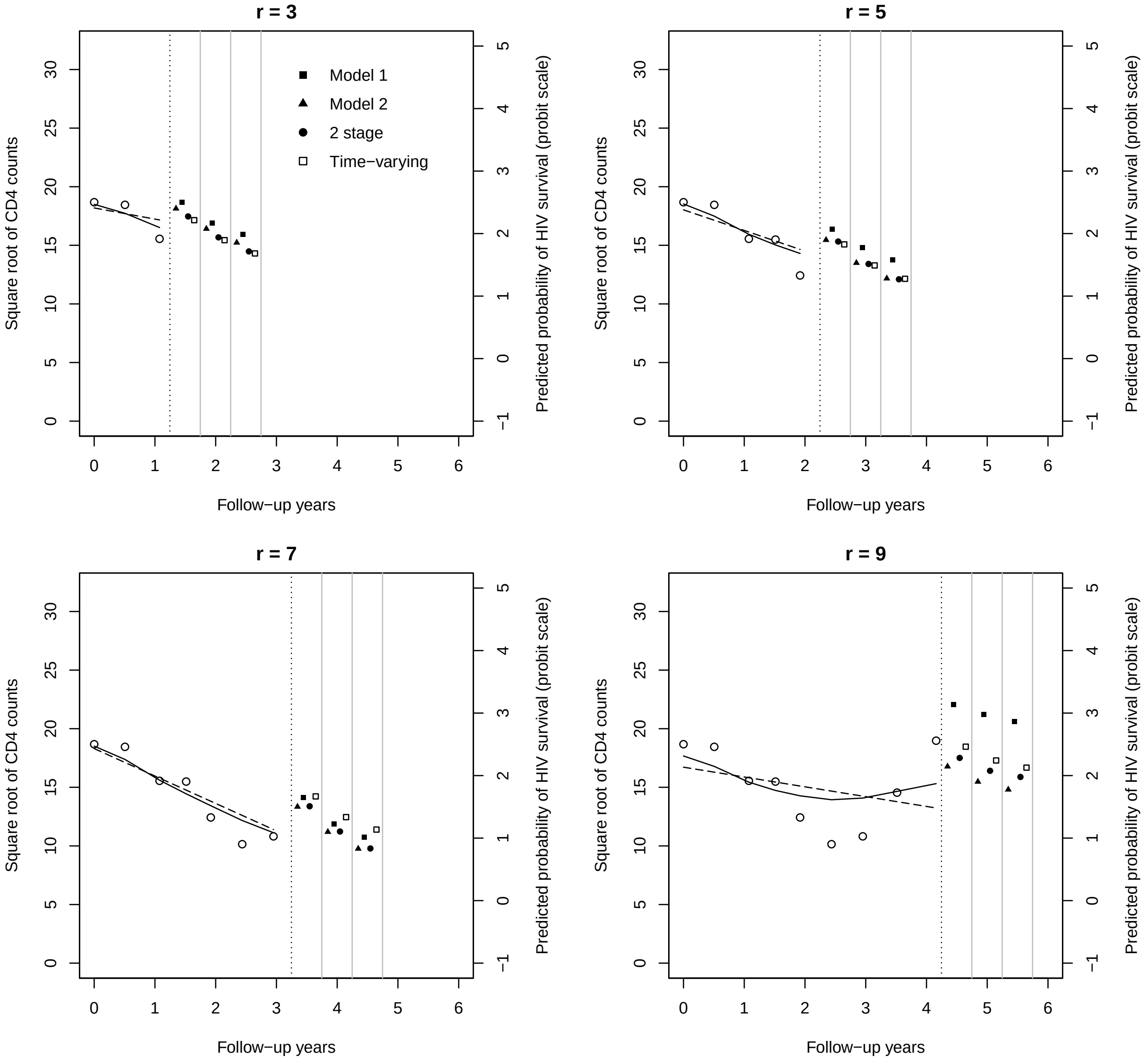

Figure 3: The fitted curve to the HERS CD4 count data by smoothing splines and non-linear least squares, assuming independence between observations within patients. t is scaled by 2093 days (the maximum follow-up days in the HERS) such that $t \in [0, 1]$.

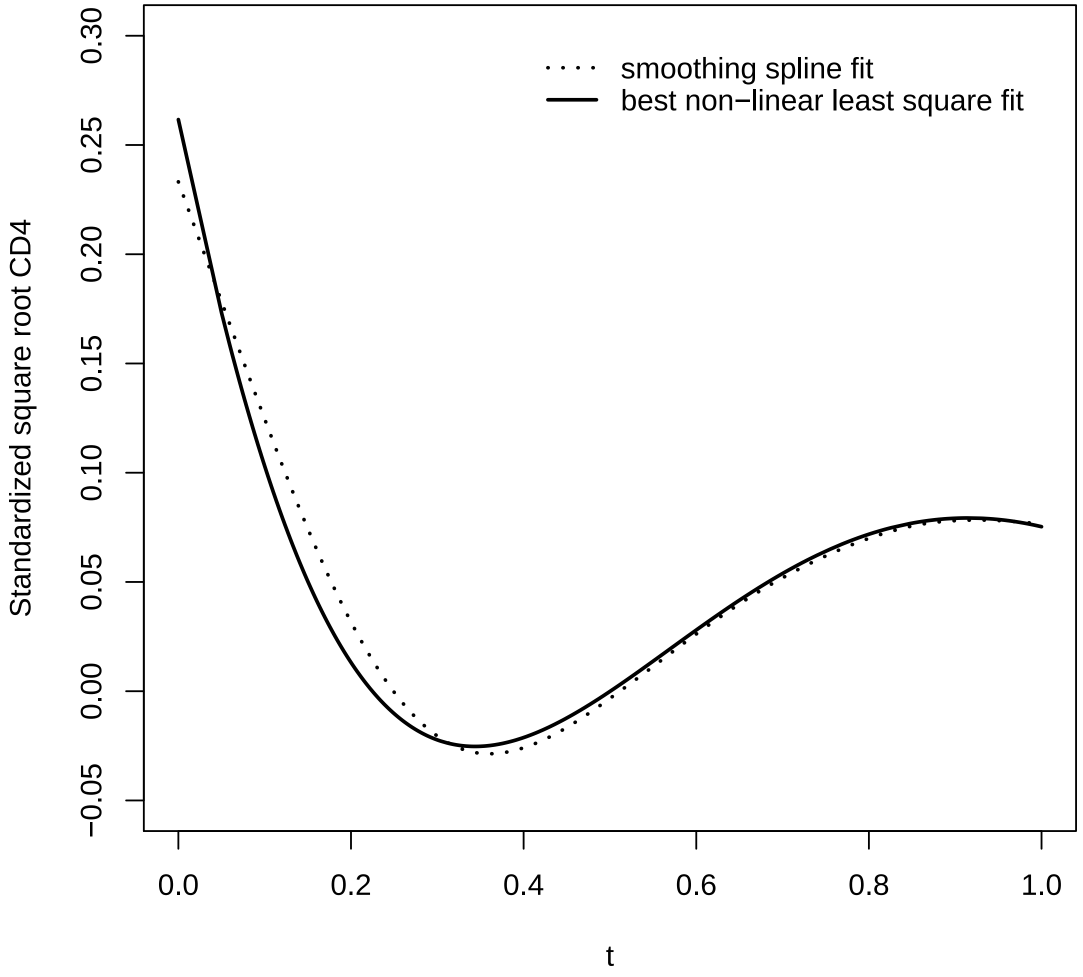

Figure 4: Selected true $m_i(t)$ given b_{i0}, b_{i1} from simulations.

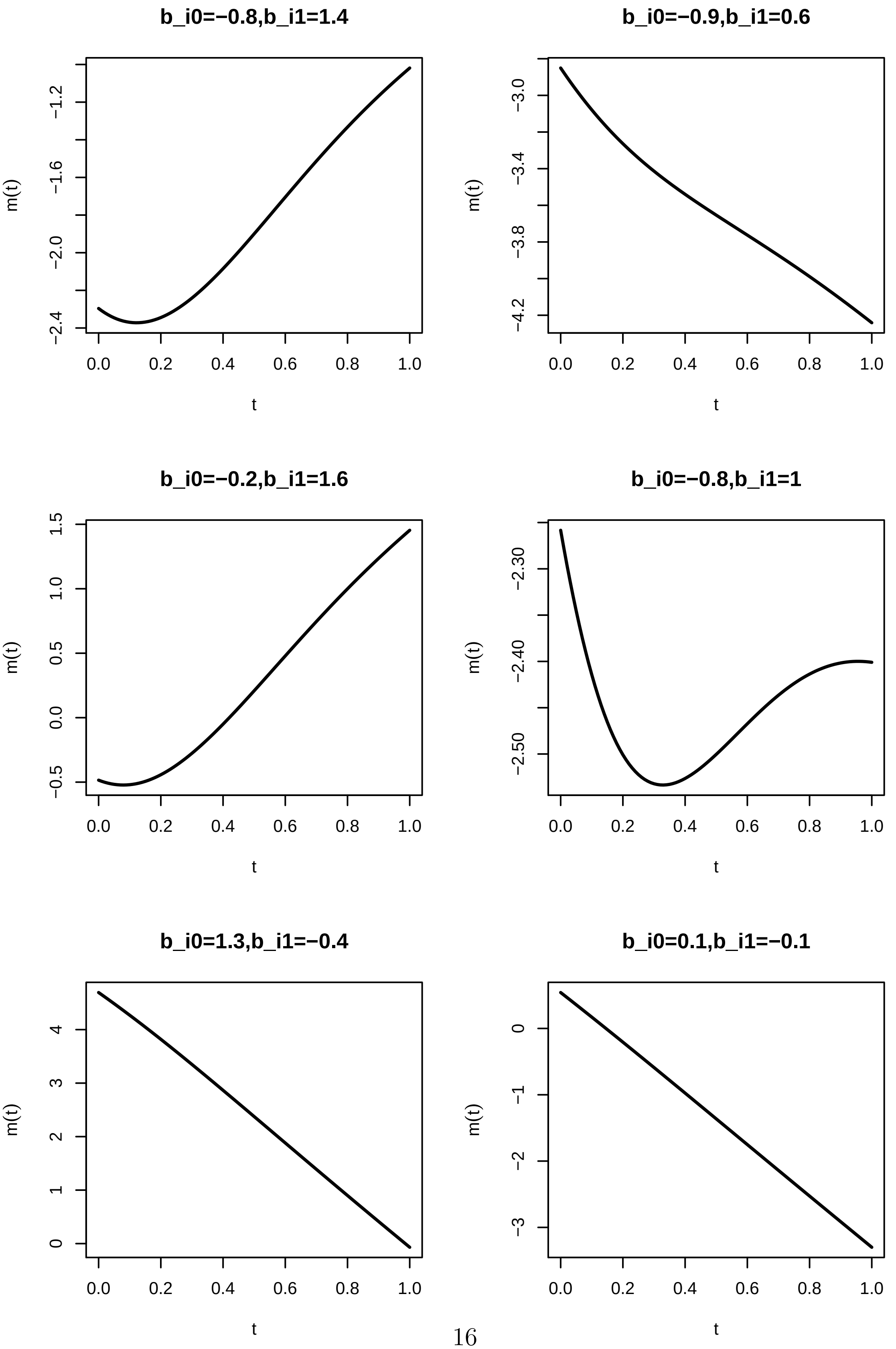

Figure 5: Mean estimates and mean squared errors for the population curve $\mu(t)$ (with grid points of 0.01 in $[0, 1]$) from 200 simulations.

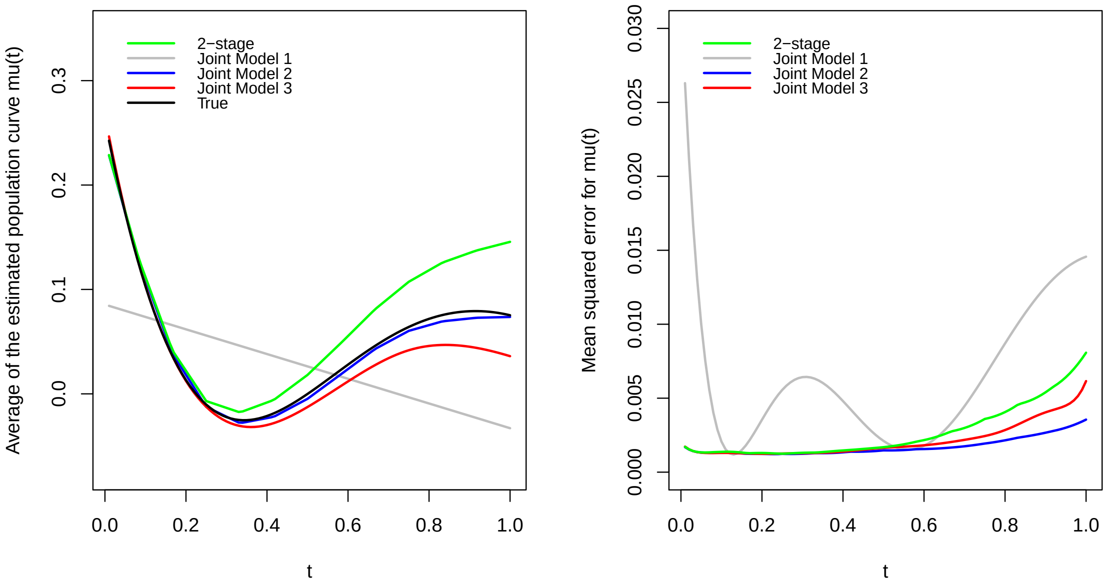

Figure 6: Boxplots of root mean squared prediction errors (RMSE) from 200 simulations for $\Delta t = 1$. Boxplot 1: RMSE from the survival model with longitudinal outcome as a time-varying covariate; Boxplot 2: RMSE from the two-stage approach; Boxplot 3-5: RMSE from Joint Models 1-3 using sample mean of $\mathbf{b}_i$; Boxplot 6-8: RMSE from Joint Models 1-3 using sample median of $\mathbf{b}_i$; Boxplot 9-11: RMSE from Joint Models 1-3 using sample mode of $\mathbf{b}_i$.

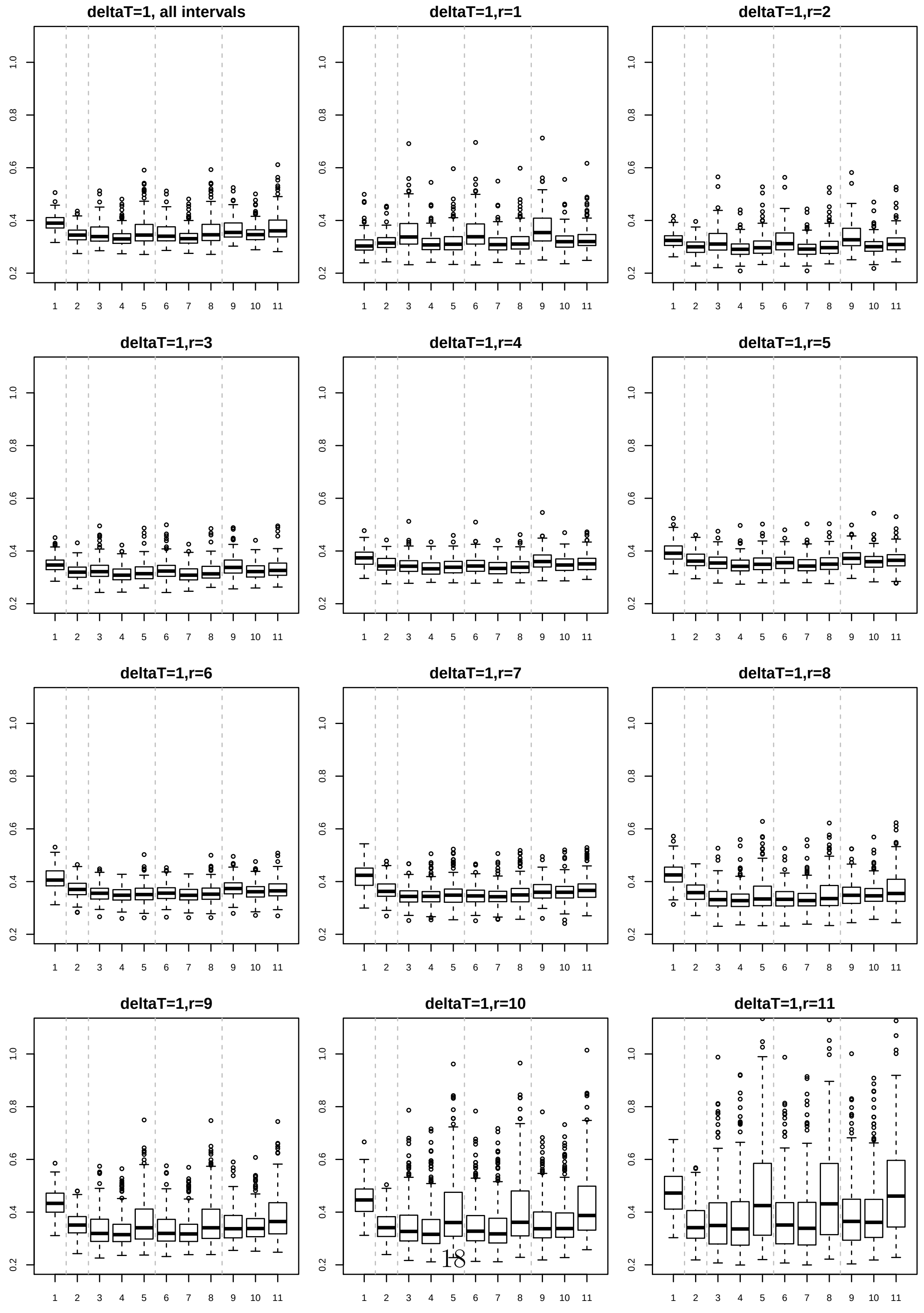

Figure 7: Boxplots of root mean squared prediction errors (RMSE) from 200 simulations for $\Delta t = 2$. Boxplot 1: RMSE from the survival model with longitudinal outcome as a time-varying covariate; Boxplot 2: RMSE from the two-stage approach; Boxplot 3-5: RMSE from Joint Models 1-3 using sample mean of $\mathbf{b}_i$; Boxplot 6-8: RMSE from Joint Models 1-3 using sample median of $\mathbf{b}_i$; Boxplot 9-11: RMSE from Joint Models 1-3 using sample mode of $\mathbf{b}_i$.

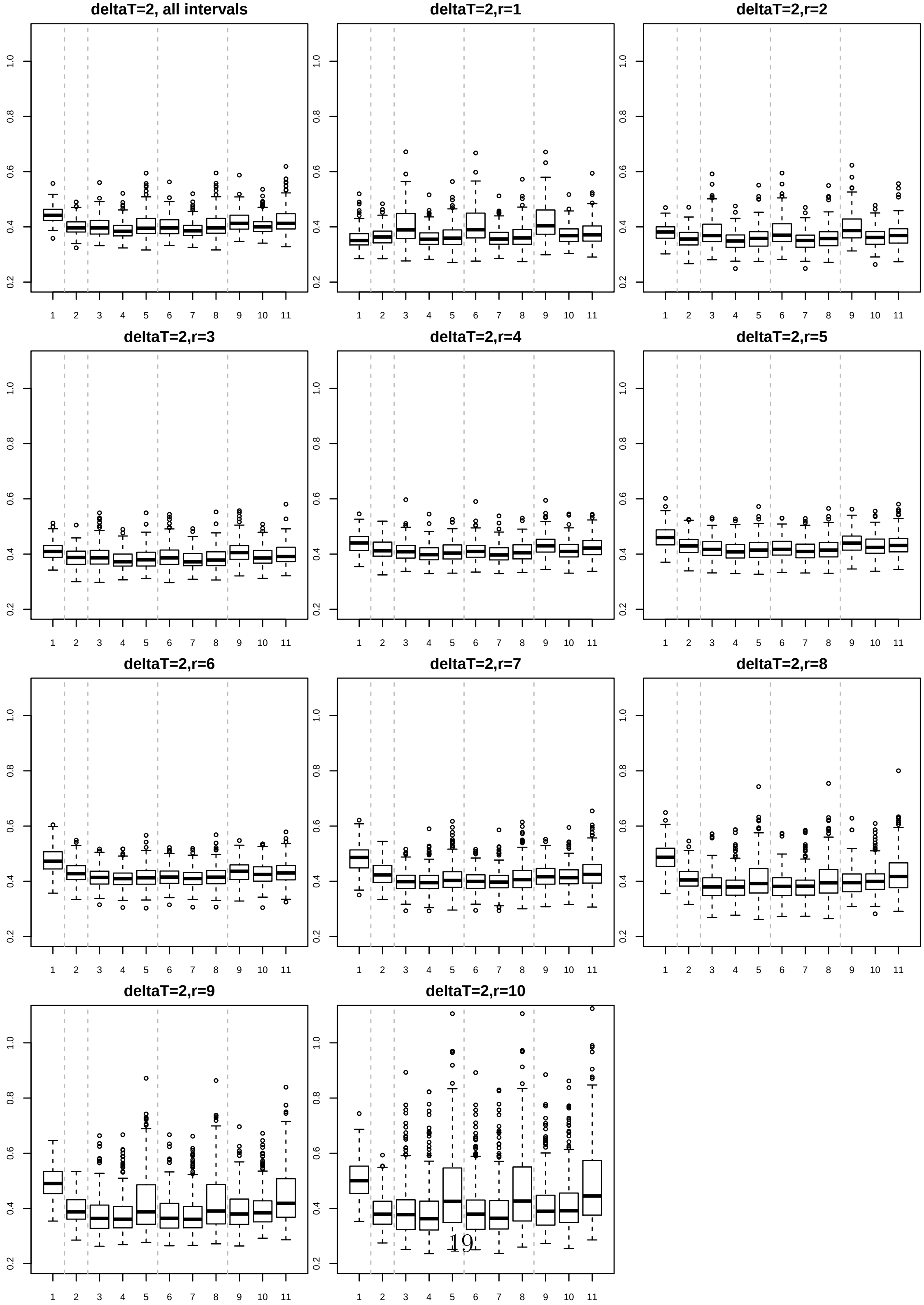

Figure 8: Boxplots of root mean squared prediction errors (RMSE) from 200 simulations for $\Delta t = 3$. Boxplot 1: RMSE from the survival model with longitudinal outcome as a time-varying covariate; Boxplot 2: RMSE from the two-stage approach; Boxplot 3-5: RMSE from Joint Models 1-3 using sample mean of $\mathbf{b}_i$; Boxplot 6-8: RMSE from Joint Models 1-3 using sample median of $\mathbf{b}_i$; Boxplot 9-11: RMSE from Joint Models 1-3 using sample mode of $\mathbf{b}_i$.

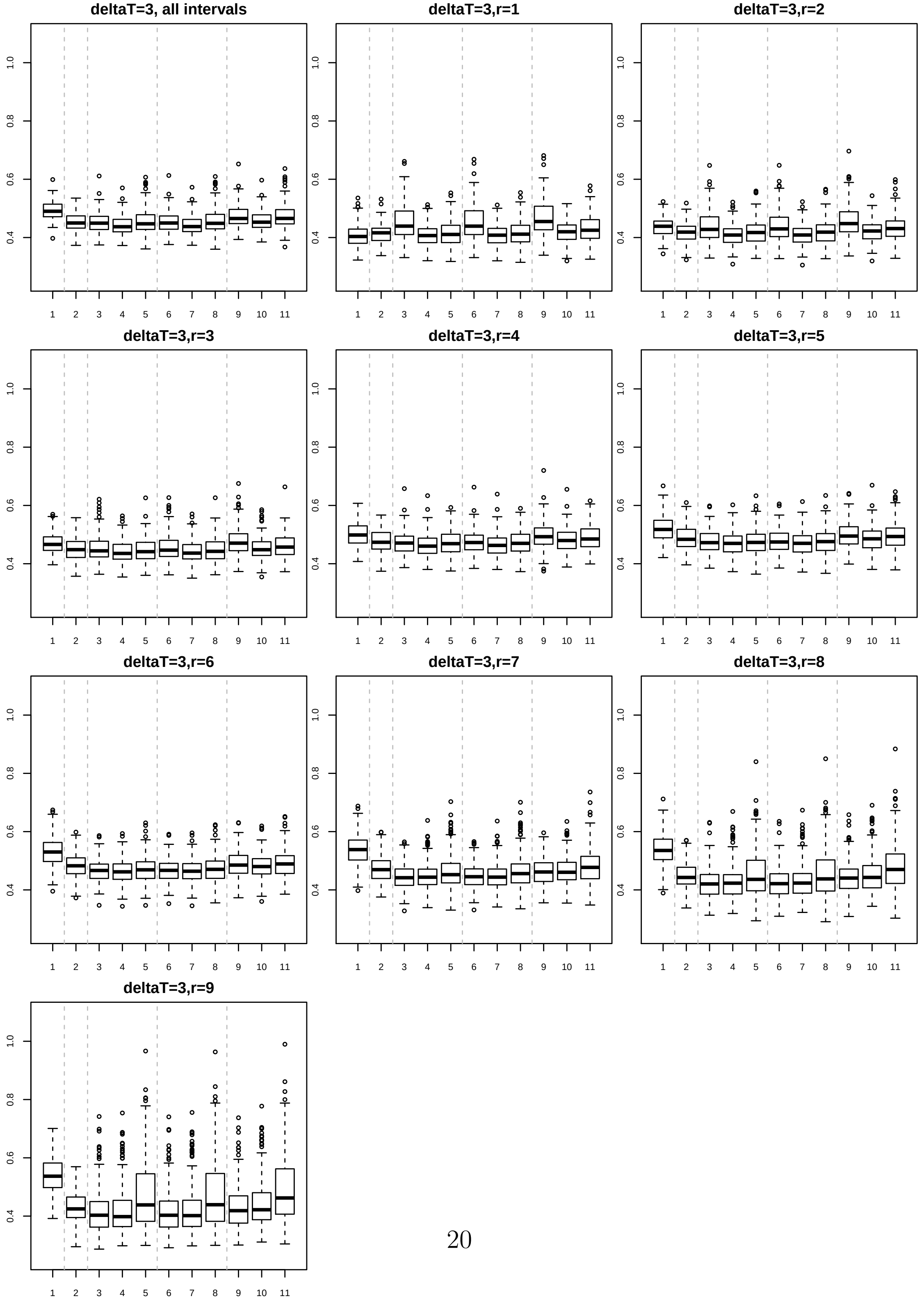

Supplement: Supplementary file 1 — Supporting info item [file SIM-36-1447-s001.zip › supplement_Barrett_Su_SIM-15-0679_R2.pdf]
